# Comparative Analysis of Muscle Transcriptome between Pig Genotypes Identifies Genes and Regulatory Mechanisms Associated to Growth, Fatness and Metabolism

**DOI:** 10.1371/journal.pone.0145162

**Published:** 2015-12-22

**Authors:** Miriam Ayuso, Almudena Fernández, Yolanda Núñez, Rita Benítez, Beatriz Isabel, Carmen Barragán, Ana Isabel Fernández, Ana Isabel Rey, Juan F. Medrano, Ángela Cánovas, Antonio González-Bulnes, Clemente López-Bote, Cristina Ovilo

**Affiliations:** 1 Departamento de Producción Animal, Facultad de Veterinaria, Universidad Complutense, Madrid, Spain; 2 Departamento de Mejora Genética Animal, INIA, Madrid, Spain; 3 Comparative Physiology Lab SGIT-INIA, Madrid, Spain; 4 Department of Animal Science, University of California Davis, Davis, California, United States of America; University of Lleida, SPAIN

## Abstract

Iberian ham production includes both purebred (IB) and Duroc-crossbred (IBxDU) Iberian pigs, which show important differences in meat quality and production traits, such as muscle growth and fatness. This experiment was conducted to investigate gene expression differences, transcriptional regulation and genetic polymorphisms that could be associated with the observed phenotypic differences between IB and IBxDU pigs. Nine IB and 10 IBxDU pigs were slaughtered at birth. Morphometric measures and blood samples were obtained and samples from *Biceps femoris* muscle were employed for compositional and transcriptome analysis by RNA-Seq technology. Phenotypic differences were evident at this early age, including greater body size and weight in IBxDU and greater *Biceps femoris* intramuscular fat and plasma cholesterol content in IB newborns. We detected 149 differentially expressed genes between IB and IBxDU neonates (*p* < 0.01 and Fold-Change > 1. 5). Several were related to adipose and muscle tissues development (*DLK1*, *FGF21* or *UBC*). The functional interpretation of the transcriptomic differences revealed enrichment of functions and pathways related to lipid metabolism in IB and to cellular and muscle growth in IBxDU pigs. Protein catabolism, cholesterol biosynthesis and immune system were functions enriched in both genotypes. We identified transcription factors potentially affecting the observed gene expression differences. Some of them have known functions on adipogenesis (*CEBPA*, *EGRs*), lipid metabolism (*PPARGC1B*) and myogenesis (*FOXOs*, *MEF2D*, *MYOD1*), which suggest a key role in the meat quality differences existing between IB and IBxDU hams. We also identified several polymorphisms showing differential segregation between IB and IBxDU pigs. Among them, non-synonymous variants were detected in several transcription factors as *PPARGC1B* and *TRIM63* genes, which could be associated to altered gene function. Taken together, these results provide information about candidate genes, metabolic pathways and genetic polymorphisms potentially involved in phenotypic differences between IB and IBxDU pigs associated to meat quality and production traits.

## Introduction

The pig is the main species for meat consumption worldwide, 43% of total produced meat comes from pigs. Most production comes from the modern European pig breeds, which have been extensively selected and show optimized productivity and efficiency [1]. In the Mediterranean basin, there is also a significant production of unique high-quality traditional pork products from local breeds. The Mediterranean breeds, also known as fatty-pig breeds, have an ancient origin, and have been reared in extensive conditions for centuries, exposed therefore to harsh environments and seasonal variations in food availability (associated with the development of a thrifty genotype [2]). These breeds are smaller in size, have not undergone intense genetic selection and are less productive than modern breeds. As a consequence of the industrialization of pork production, three-quarters of the traditional breeds are extinct or marginalized [3]. The exception is the Iberian pig, the most representative Mediterranean traditional breed, which has an important commercial value based on high quality dry-cured products in terms of consumers’ health and acceptance [4].

Peculiarities in Iberian pig metabolism drive its valued meat properties; Iberian pigs are characterized by higher fat deposition, fat desaturation and food intake [5, 6], as well as by higher circulating leptin levels in plasma [7] than lean pigs, suggesting a syndrome of leptin resistance. Moreover, the Iberian pig is also considered an amenable and robust biomedical model for obesity and associated cardiometabolic diseases since, when provided high levels of food, the animals are prone to the development of dyslipidemias, metabolic syndrome and type-2 diabetes [8]. On the other hand, as observed in other traditional breeds, productive performance is considerably lower than that of highly selected modern breeds. To improve reproductive and growth performances and primal cuts yield, in the last decades Duroc breed was introduced as terminal sire cross. Recently, Spanish law has accepted and regulated the use of Iberian X Duroc pigs to obtain “Iberian” products.

However, the introduction of Duroc genetics is associated with a decrease in meat quality, mainly determined by a decrease in intramuscular fat (IMF) and monounsaturated fatty acids (MUFA) contents [9]. Intramuscular fat content and fatty acid composition are the main factors affecting meat quality and are highly dependent on genetic type and diet [10]. Intramuscular fat content is determined both by number and size of adipocytes within muscle fibers. During prenatal development and immediately after birth, preadipocyte differentiation is a very active process that slow down with animal growth [11]. Later in growth adipocyte hypertrophy is the most important issue affecting IMF content, although hyperplasia is maintained in the adult animal to a lesser extent [12]. Thus, birth is a critical time-point to investigate the adipocyte differentiation process. On the other hand, IMF composition and fatty acids profile depend on lipogenesis and fatty acids metabolism. It has been reported that breed affects adipogenesis, lipogenesis and their timing, as well as the expression patterns of adipocyte differentiation-related genes [13]. In this sense, Iberian pig is considered a more precocious breed than Duroc pig [14].

Due to the influence of the genetic background on productive and meat quality traits, research in the past few decades has been focused on understanding the genetic basis of cell growth and development, myogenesis and metabolism [15]. Recently, new interest has arisen towards the understanding of genetic mechanisms underlying lipid synthesis and accumulation, due to its importance in meat quality [13]. Different approaches such as candidate gene expression studies or cDNA microarray analysis have been used to investigate genetic aspects of target parameters. Some studies based on the microarray technology investigated transcriptome differences among Iberian pig and Large White or Duroc pig in endocrine tissues [16] and between Iberian and Iberian X Duroc crossbred pigs in *Longissimus dorsi* muscle [14].

Currently, the availability of the RNA-Seq technology has allowed the assessment of global changes in transcriptome of a number of species including pigs [15], because of its greater accuracy and reproducibility than microarray technology [17, 18]. RNA-Seq allows measuring not only gene expression, but also examining genome structure identifying SNP and other structural variation such as indel and splice variants. Some applications of this technology include transcript quantification, allele-specific expression, novel transcript discovery or single nucleotide polymorphism (SNP) discovery [19]. In pigs, several RNA-Seq studies have been carried out for assessing differences in the transcriptome of muscle, fat, liver or hypothalamus among breeds or phenotypically extreme individuals within a breed for characters of interest [15, 20, 21]. The RNA-Seq technology is still scarcely applied to the Iberian breed, with studies comprehending the assessment of phenotypically extreme individuals for fatty acids composition [22] or the exploration of gonad transcriptome in Iberian and Large White pigs [23]. However, to the best of our knowledge, there are not RNA-Seq technology-based studies focused on genetic differences between Iberian and other breeds aimed at improving meat quality and productive traits.

Meat quality in Iberian pigs is of special interest for carcass cuts used in the dry curing industry such as the loin and the ham. A previous study assessed transcriptomic differences between pure Iberian and Duroc-crossbred Iberian pigs using microarray technology in the loin [14], but no information on ham muscles transcriptome exists. It is well known that different muscles differ in developmental timing, metabolic and physicochemical properties, including different responses to exercise [24]. *Biceps femoris* (BF) muscle is the biggest muscle in the ham and shows higher oxidative capacity, and lower drip loss than *Longissimus dorsi* muscle [25, 26]. Moreover, important differences exist regarding the IMF content of both muscles. Karlsson et al. (1993) reported higher IMF content in LD muscle in Yorkshire pig breed, whilst the opposite was reported for Iberian pigs, where BF showed remarkably greater IMF content than several others carcass muscles [27]. Also, transcriptomic and proteomic comparisons between muscles showed important functional differences, with 15–30% of proteome differing between LD and BF [24, 28].On the other hand, transcriptomic studies performed sequentially along early development suggest the perinatal as a critical period to study genes affecting muscle and adipose cells growth and muscle fiber differentiation [20, 29], in agreement with the tissue differentiation timing commented previously. Moreover, environmental effects are minimized at this time point.

Hence, in agreement with previous considerations, the present study was carried out to study the BF muscle of newborn piglets in IB and in the IBxDU cross, aiming to: 1) Verify whether phenotypic differences are evident from the very early developmental stages (newborns) in these closely related populations; 2)Evaluate changes in gene expression *in BF* muscle that may be responsible for the observed phenotypic differences and identify pathways and networks in which those genes are involved; 3) Identify transcription factors affecting gene expression in order to establish potential new candidate genes affecting productive parameters and meat quality; 4) Identify structural variants in these candidate genes, potentially involved in the observed expression differences.

These results are useful for the understanding of genetic pathways affecting pork production and may be also of translational value for the understanding of ethnic differences in obesity and associated disorders in lipid metabolism in human medicine.

## Materials and Methods

### Ethics statement

Animal manipulations were done in compliance with the regulations of the Spanish Policy for Animal Protection RD1201/05, which meets the European Union Directive 86/609 about the protection of animals used in research. The experiment was specifically assessed and approved (report CEEA 2010/003) by the INIA Committee of Ethics in Animal Research, which is the named Institutional Animal Care and Use Committee (IACUC) for the INIA.

### Animals and sample collection

Ten pure Iberian sows raised in the same commercial farm were employed at their third gestation cycle. All females were managed in the same conditions. Five sows were mated to Iberian boars and five to Duroc boars. At birth, nine pure Iberian (IB) and 10 Iberian x Duroc (IBxDU) male piglets were randomly selected from the ten litters (two from each litter excepting one litter providing just one Iberian male). Blood samples were collected from newborns in sterile heparin blood vacuum tubes (Vacutainer Systems Europe, Meylan, France). Immediately after recovery, the blood was centrifuged at 1500g for 15 min and the plasma was separated and stored into polypropylene vials at −20°C until assayed for determination of glucose and lipids metabolism-indicating parameters. After blood collection, piglets were slaughtered. Several body development measures were obtained with a measure-tape: total body length (from the rostral edge of the snout to the tail insertion), ham length (from the anterior edge of the *Symphysis pubica* to the *articulatio tarsi)*, total length of anterior and posterior limbs (from the distal edge of the hooves to the proximal edge of the *scapula* or *Symphysis pubica*, respectively) and thoracic, abdominal and ham circumferences. Carcasses were weighted and samples from BF muscle were vacuum-packed in low-oxygen permeable film and kept frozen at –20°C until fatty acid composition analysis. Prior to fatty acid analysis, muscle samples were freeze dried for two days in a lyophilizer (Lyoquest, Telstar, Tarrasa, Spain) and grounded in a Mixer Mill MM400 (Retsch technology, Haan, Germany) until muscle was completely powdered. For transcriptomic analysis, BF samples were immediately frozen in liquid nitrogen and maintained at –80°C until RNA extraction.

The metabolic status of the newborn piglets was evaluated. Glucose, fructosamine, triglycerides, total cholesterol, high-density lipoprotein cholesterol (HDL-c) and low-density lipoprotein cholesterol (LDL-c) plasmatic levels were measured with a clinical chemistry analyzer (Saturno 300 plus, Crony Instruments s. r. l., Rome, Italy).

### Tissue composition analysis

*Biceps femoris* muscle IMF content was quantified using the method proposed by Segura and López-Bote [30] based on gravimetrical determination of lipid content. Fatty acid methyl esters (FAMEs) were identified by gas chromatography as described by López-Bote *et al* [31] using a Hewlett Packard HP-6890 (Avondale, PA, USA) gas chromatograph equipped with a flame ionization detector and a capillary column (HP-Innowax, 30 m × 0.32 mm i.d. and 0.25 μm polyethylene glycol-film thickness). Results were expressed as grams per 100 grams of detected FAMEs.

### Transcriptomic analysis

#### RNA extraction

A total of 12 animals were randomly selected to perform transcriptomic analysis, representing all available litters (6 animals of each genetic type). Total RNA was extracted from 50–100mg samples of *BF* muscle using the RiboPure TM of High Quality total RNA kit (Ambion, Austin, TX, USA) following the manufacturer’s recommendations. RNA was quantified using a NanoDrop-100 spectrophotometer (NanoDrop Technologies, Wilmington, DE, USA). The quality of the RNA was evaluated using the RNA Integrity Number (RIN) value from the Agilent 2100 Bioanalyzer device (Agilent technologies, Santa Clara, CA, USA). The RIN values ranged from 7.5 to 9.8.

#### Library construction and RNA sequencing

Sequencing libraries were made using the mRNA-Seq sample preparation kit (Illumina Inc., Cat. # RS-100-0801) according to manufacturer’s protocol. Each library was sequenced using TruSeq SBS Kit v3-HS, in paired end mode with the read length 2x76bp on a HiSeq2000 sequence analyzer (Illumina, Inc). Images from the instrument were processed using the manufacturer’s software to generate FASTQ sequence files.

#### Mapping and assembly

Sequence reads were analyzed using CLC Bio Genomic workbench software 7.0 (CLC Bio, Aarhus, Denmark). Quality control analysis was performed using the NGS quality control tool, which assesses sequence quality indicators based on the FastQC-project (http://www.bioinformatics.babraham.ac.uk/projects/fastqc/). Quality was measured taking into account sequence-read lengths and base-coverage, nucleotide contributions and base ambiguities, quality scores as emitted by the base caller and over-represented sequences [32]. All the samples analyzed passed all the QC parameters having the same length (76 bp), 100% coverage in all bases, 25% of A, T, G and C nucleotide contributions, 50% GC on base content and less than 0.1% over-represented sequences. A hierarchical clustering of the samples was also performed. One IBxDU pig was discarded for further analysis because the sample deviated largely from the expected grouping in the clustering analysis, probably due to RNA sampling or processing problems. Sequence paired-end reads (76bp) were assembled against the annotated Sscrofa10.2 reference genome (http://www.ncbi.nlm.nih.gov/genome/?term=sus+scrofa) using the genome, annotated genes and mRNA tracks. Data was normalized by calculating the ‘fragments per kilo base per million mapped reads’ (FPKM) for each gene [33].

#### Differential expression analysis

The statistical analysis was performed using the total exon reads as expression values by the Empirical analysis of differential gene expression tool. This tool is based on the EdgeR Bioconductor package [34] and uses count data (i.e. total exon reads) for the statistical analysis. Genes were filtered according to two criteria: a minimum mean group expression greater than 0.5 FPKM in at least one group and a Fold-Change (FC) of the expression differences between IB and IBxDU groups equal or higher to 1.5. Finally, those genes with a *p* ≤ 0.01, corresponding to a false discovery rate (FDR) value ≤ 0.23, were considered as differentially expressed (DE).

#### Systems biology study

The biological interpretation of the DE genes observed in BF muscle was performed using three complementary approaches, in order to identify enriched GO terms, pathways and networks involving the DE genes, and potential regulators causing the observed changes in gene expression.

The enrichment analysis was carried out using the Wilcoxon test tool in the ConsensusPathDB database [35], available at the Max Plank Institute website, which provides batch enrichment analyses to highlight the most relevant GO terms associated to a gene list. Both, the list of genes overexpressed in IB and IBxDU were used. Functional terms with *p-*values lower than 0.05 were considered enriched in the annotation categories. The *p-*values are corrected for multiple testing using the FDR method and are presented as *q*-values.

Additionally, Ingenuity Pathway Analysis, (IPA)(Ingenuity Systems, Qiagen, California)software was employed to identify and characterize biological functions, gene networks and canonical pathways affected by the DE genes.

Regulatory transcription factors (TRF), which could potentially affect the DE genes in the dataset were also studied by following complementary approaches. First, Regulatory Impact Factors (RIF1 and RIF2) metrics [36, 37] were calculated for the whole set of DE genes obtained conditional on genetic type (149 genes). RIF1 assigns an extreme score to those TRF that are consistently most differentially co-expressed with the highly abundant and highly DE genes; RIF2 assigns an extreme score to those TRF with the most altered ability to act as predictors of the abundance of DE genes. Candidate TRFs in pigs were obtained from Animal TFDB (http://www.bioguo.org/AnimalTFDB/BrowseAllTF.php?spe=Susscrofa). A total of 1038 TRF were retrieved. Among them, 723 showed expression values greater than 0.5 FPKM in at least one experimental group and thus, were used in the RIFs metrics approach.

The RIF1 and RIF2 values were computed for the *i* ^th^ TRF as follows:

$${RIF1}_{i}=\frac{1}{n_{de}}\sum_{j=1}^{j=n_{de}}\;{\hat{a}}_{j}x{\hat{d}}_{j}{\left({r1}_{ij}-{r2}_{ij}\right)}^{2}\;\text{and}$$

$${RIF2}_{i}=\frac{1}{n_{de}}\sum_{j=1}^{j=n_{de}}[{({{e1}_{j}{rx1}_{ij})}^{2}-\left({e2}_{j}x{r2}_{ij}\right)}^{2}]$$

where *n*_*de*_ is the number of DE genes, *a*_j_ and *d*_*j*_ the estimated average expression and differential expression of the *j* ^*th*^ DE gene, *r1*_*ij*_ and *r2*_*ij*_ the co-expression correlation between the *i* ^*th*^ TRF and the *j* ^*th*^ DE gene in each one of the genetic types and being*e1*_*j*_ and *e2*_*j*_ the expression of the *j* ^*th*^ gene in each genetic type [38]. Both RIF measures for each analyzed TRF were transformed to standardized *z*-scores by subtracting the mean and dividing by its standard deviation. We identified relevant TRF as those with extreme RIF z-scores according to the corresponding confidence intervals (CI) calculated by bootstrap. In each iteration of bootstrapping, a set of n_de_ = 149 genes were randomly selected from the 11392 expressed genes, and the RIF1 and RIF2 z-scores of the 723 TRF were calculated. The procedure was repeated 10,000 times for each scenario to obtain the corresponding 95 and 99% CI intervals of both z-scores. Complementarily, IPA software was employed to identify and characterize potential regulators using two different tools, the *upstream regulators* and the *regulators* tools. Both of them identify known regulators that may be affecting expression of the dataset of DE genes. IPA-identified regulators include genes, but also other molecules as drugs. Thus, out of the identified regulators, only genes that were also included in the RIFs metrics candidate TRF list (which consisted of 723 TRF) were considered (genes included in the animal TFDB and with expression values higher than 0.5 FPKM in at least one experimental group). Using the information obtained from the TRF study, an additional search for enriched pathways and networks was carried out with IPA software considering both, DE genes and TRF.

#### Structural variants analysis

A search of structural variants was performed by pooling the reads coming from all animals in each genetic type, and comparing the variants found in each group. The probabilistic variants detection tool (CLC Bio Genomic workbench) was used to perform the variant calling analysis. Single-end read alignments were not ignored. The minimum coverage in a locus to be considered was set up as 10 and the variant probability as 90. The variant probability parameter defines how good the evidence has to be at a particular site for the tool to report a variant at that location. Specifically, the variant probability threshold set as 90 means that any candidate variation in the genome must have a probability lower than 0.1 (1–0.9) of being the same as the reference sequence, to be considered as a variant. Variants with an allele frequency under 5% and/or coverage under 30 reads for the general variant analysis and under 10 reads in the candidate genes variant analysis were not considered. Variants were considered to be potentially fixed (frequency greater or equal to 90%) or segregating (frequency lower than 90%).

The variants identified in genes corresponding to transcription factors (i.e. *EGR2*, *FOS*, *FOXO1*, *FOXO3*, *IRF1*, *STAT5B*, *HOXA9*, *ATF4*, *TP53*, *NOR-1*, *ABRA*, *ATF3* and *PPARGC1B*) were functionally evaluated using the variant effect predictor (VEP) tool from Ensembl (www.ensembl.org/info/docs/tools/vep/) which includes information about amino acid change localization and consequences, affected transcripts, and SIFT [39] and PolyPhen [40] scores.

### Results validation by RT qPCR

RNA obtained from the 11 animals under study was employed to perform the technical validation of the differential expression of some genes that were either upregulated in IB, upregulated in IBxDU or not DE between genetic types. This technical validation was performed by studying the Pearson correlation between the expression values obtained from RNA-Seq data (FPKM) and the normalized gene expression data obtained by RT qPCR. Moreover, RNA obtained from all the available animals (9 IB and 10 IBxDU) was used to quantify expression differences of such genes.

First-strand cDNA synthesis was carried out with Superscript II (Invitrogen, Life Technologies, Paisley, UK) and random hexamers in a total volume of 20 μl containing 1 μg of total RNA and following the supplier’s instructions.

The expression of 9 genes was quantified by qPCR. Primer pairs used for quantification were designed using Primer Select software (DNASTAR, Wisconsin, USA) from the available GENBANK and/or ENSEMBL sequences, covering different exons in order to assure the amplification of the cDNA. Sequence of primers and amplicon lengths are indicated in S1 Table. Standard PCRs on cDNA were carried out to verify amplicon sizes. Quantification was performed using SYBR Green mix (Roche, Basel, Switzerland) in a LightCycler480 (Roche, Basel, Switzerland), following standard procedures Data were analyzed with LightCycler480 SW1.5 software (Roche, Basel, Switzerland). All samples were run in triplicate and dissociation curves were carried out for each individual replicate. Single peaks in the dissociation curves confirmed the specific amplification of the genes. For each gene, PCR efficiency was estimated by standard curve calculation using four points of cDNA serial dilutions. Mean Cp values were employed for the statistical analyses of differential expression. Stability of four endogenous genes (i. e. *GAPDH*, *B2M*, *TBP* and *ACTB)* was calculated using Genorm software [41] The *TBP* and *ACTB* genes were selected as the most stable endogenous genes to normalize the data. The qPCR expression data normalization was performed using normalization factors calculated with Genorm software. Relative quantities were divided by the normalization factors, which were the geometric means of the two reference genes quantities.

### Statistical analyses of tissue composition and qPCR expression quantification

Phenotypic data were analyzed as a completely randomized design using the general linear model (GLM) procedure using SAS version 9.2 (SAS Inst. Inc., Cary, NC; 2009). The mean and genetic type were considered as systematic effects, and residual effects as random. Carcass weight was used as covariate when it was significant and removed from the model when it was insignificant. The animal was the experimental unit for all analysis. The results were considered to be significant at *p-*value<0.05.

Statistical analysis of gene expression data was carried out following the method proposed by Steibel *et al* [42] which consists of the analysis of cycles to threshold values (Cp), for the target and endogenous genes using a linear mixed model. The following model was used for analyzing the joint expression of the target and control genes in different tissues:

$$y_{gikr}={TG}_{gi}+B_{gk}+D_{ik}+e_{gikr}$$

where, $y_{gijkr}\;=-{log}_{2}\left(E_{g}^{{-Cp}_{gijkr}}\right)$, *E*_*g*_ is the efficiency of the PCR of *g*th gene, *Cp*_*gikr*_ is the value obtained from the thermocycler software for the *g*th gene from the *r*th replicate in a sample collected from the *k*th animal of the *i*th genetic type, *TG*_*gi*_ is the specific effect of the *i*th genetic type on the expression of gene *g*th, *B*_*gjk*_ is specific random effect of the *k*th pig on the expression of gene *g*th, *D*_*ijk*_ is a random sample-specific effect common to all the genes, and *e*_*gikr*_ is a residual effect.

To test differences in the expression rate of genes of interest (*diff*_*TG*_) between classes normalized by the endogenous genes, different contrasts were performed between the respective estimates of *TG* levels. Significance of *diff*_*TG*_ estimates was determined with the *t* statistic. To obtain FC values from the estimated *diff*_*TG*_ values, the following equation was applied: $FC\;=\;2^{{-diff}_{TG}}$

*P-*values<0.05 were considered statistically significant.

To validate the global RNA-Seq results, the concordance correlation coefficient (CCC) [43] was calculated between the FC values estimated in BF muscle from RNA-Seq and qPCR expression measures for the 9 genes analyzed by the two technologies (RNA-Seq and qPCR).

## Results and Discussion

### Phenotypic differences between genetic types

The results obtained in the present study constitute the first assessment of phenotypic differences between IB and IBxDU piglets at birth. There are several studies evaluating phenotypic differences between both genotypes at weaning or adulthood [9, 14, 44–46]. Pure Iberian and crossbred piglets were slaughtered at birth at an average of 1.2 and 1.8 kg live weight, respectively (SEM = 0.06). Genetic type affected all the carcass phenotypic parameters: IBxDU neonates were bigger and heavier (*p* < 0.001) than IB newborns (Table 1), reflecting previously reported differences in the same traits in adult animals [45, 46]. Birth is an interesting sampling time to study differences in metabolism and growth, in which environmental influences are minimized, but maternal effects may have to be considered. Unfortunately no information regarding the employed sows’ phenotype was available, but all of them originated from the same commercial Iberian population and were checked for breed purity by genotyping several markers usually employed for breed origin authentication, showing homocygosity for Iberian alleles. Moreover age and productive cycle were also homogeneous among them as indicated in the methods section, and thus, we don’t expect that potential sow effects may interfere with the observed between-genotype differences regarding piglet’s phenotype or transcriptome.

**Table 1:** Carcass, *Biceps Femoris* and metabolism phenotypic characteristics in IB and IBxDU piglets.

	Genetic type		SEM^3^	*p-*value
	IBxDU^1^	IB^2^		
Carcass characteristics				
Carcass weight, kg	1.41	0.96	0.05	0.0005
Ham weight, kg	0.16	0.11	0.00	0.0008
Total body lenght, cm	40.20	35.50	0.31	0.0004
Ham lenght, cm	7.45	6.33	0.12	0.0009
Forelimb lenght, cm	12.35	10.83	0.12	0.0042
Hind limb lenght, cm	15.95	13.83	0.12	0.0016
Torax circumference, cm	25.15	22.06	0.14	0.0010
Abdomen circumference, cm	18.90	17.28	0.19	0.0486
Ham circumference, cm	12.55	10.89	0.15	0.0020
Lipid and glucose metabolism-related plasma indicators				
Cholesterol, mg/dl	62.19	102.36	5.60	0.0030
Fructosamine, mg/dl	169.70	133.67	10.37	0.1009
Glucose, mg/dl	132.40	123.44	10.80	0.6839
LDL^4^, mg/dl	42.16	45.82	4.40	0.4496
HDL^5^, mg/dl	22.38	41.20	4.25	0.0176
Triglycerides, mg/dl	30.00	76.67	5.11	0.0003
*Biceps femoris* muscle fatty acids composition (g/100 g total fatty acids)				
IMF^6^, %	1.72	2.21	0.09	0.0142
C12:0	0.65	0.58	0.03	0.2321
C14:0	2.57	2.32	0.12	0.3189
C15:1	1.28	1.18	0.06	0.3762
C16:0	25.90	25.44	0.19	0.2379
C16:1 n-9	1.90	2.09	0.05	0.3854
C16:1 n-7	5.38	4.57	0.21	0.0773
C17:0	1.69	1.44	0.07	0.0814
C17:1	0.91	0.81	0.06	0.3858
C18:0	10.85	9.96	0.25	0.1014
C18:1 n-9	23.80	25.82	0.56	0.0921
C18:1 n-7	6.15	5.69	0.18	0.2163
C18:2 n-6	7.31	9.17	0.60	0.1395
C20:1 n-9	0.53	0.53	0.01	0.9701
C20:2 n-6	0.41	0.40	0.03	0.8331
C20:3 n-6	0.62	0.55	0.02	0.0293
C20:4 n-6	6.31	5.99	0.20	0.4264
C22:1 n-9	1.21	1.08	0.05	0.1956
C22:4 n-6	1.58	1.27	0.09	0.0925
C22:5 n-3	0.48	0.50	0.02	0.5405
C22:6 n-3	0.67	0.62	0.02	0.1959
∑SFA^7^	41.66	39.74	0.42	0.0350
∑MUFA^8^	41.01	41.77	0.40	0.3530
∑PUFA^9^	17.34	18.49	0.50	0.2639
UI^10^	96.20	97.79	0.90	0.3904
∑n-3^11^	1.77	1.67	0.04	0.1940
∑n-6^12^	15.56	16.82	0.48	0.2089
∑n-6/∑n-3	8.78	10.17	0.30	0.0319
∑MUFA/∑SFA	0.99	1.05	0.02	0.0659

^1^ IBxDU = Iberian x Duroc crossbred pigs (n = 10)

^2^ IB = Purebred Iberian pigs (n = 9)

^3^SEM = Standard error of the mean

^4^LDL = Low density lipoproteins

^5^HDL = High density lipoproteins

^6^IMF = Intramuscular fat

^7^ΣSFA = Sum of saturated fatty acids

^8^ΣMUFA = Sum of monounsaturated fatty acids

^9^ΣPUFA = Sum of polyunsaturated fatty acids

^10^UI = Unsaturation index = 1 × (% monoenoics) +2 × (% dienoics) +3 × (% trienoics) +4 × (% tetraenoics) +5 × (% pentaenoics) +6 × (% hexaenoics) [126]

^11^Σn3 = Sum of n-3 fatty acids

^12^Σn6 = Sum of n-6 fatty acids

The assessment of differences in glucose and lipids metabolism (Table 1) showed that purebred IB piglets have greater plasma levels of total and HDL cholesterol, and triglyceride than IBxDU neonates. These differences at birth are concordant with the similar differences previously found between purebred Iberian and lean crossbred (Large White x Landrace x Pietrain) fetuses [47]. Cholesterol is of vital importance for the offspring as a key constituent of cell membranes and the precursor of hormones and metabolic regulators [48, 49]. Placental and fetal tissues have the capacity for *de novo* cholesterol synthesis [50] but the high demand from the fetuses makes the transport of maternal cholesterol through the placenta necessary [51, 52]. Triglycerides are also indispensable as a major source of energy for the developing fetus and are also transferred from maternal circulation [53, 54]. Previous studies have found that adequate availability of cholesterol and triglycerides is even more critical in fatty-pigs breeds [55], which have higher values of plasma lipids indexes than lean breeds [47].

These results reinforce that genetic differences between fatty-pigs and lean breeds are established from prenatal stages and, together with previous results, may also give evidence of a genetic predisposition for lipid metabolism alterations in the Iberian breed. The same findings regarding plasma cholesterol and triglycerides levels have been reported in humans with familial combined hyperlipidemia, the most common genetic form of hyperlipidemia in human [56, 57] and in the Rapacz familial hypercholesterolaemic swine model.

Regarding the IMF content and composition in BF (Table 1), IB showed almost 30% higher IMF content than IBxDU piglets (*p* = 0.014). The genetic type affected IMF composition as well, IB pigs showing greater ∑n-6/∑n-3 ratio (*p* = 0.031) (mainly due to greater proportion of C18:2 n-6), and lower ∑SFA (saturated fatty acids) content (*p* = 0.035). Also, a trend for a higher oleic acid content was observed in IB pigs (*p* = 0.092). As reported in previous studies, crossing with Duroc sires decreased IMF concentration, in agreement with differences observed in adult pigs [9, 14].

The differences observed between IB and IBxDU in parameters such as body size and weight, lipid metabolism-related indicators or IMF were surprising taking in account the early stage of development. This highlights the importance of improving the knowledge on molecular aspects responsible for such phenotypic differences at very early ages with the dual purpose of improving production in local breeds with distinctive products and providing adequate models for human diseases.

### Identification of differentially expressed genes by RNA-Seq analysis

An average of approximately 79 million sequence reads was obtained for each individual sample; these were assembled and mapped to the annotated Sscrofa10.2 genome assembly (22,861 genes). In all samples, 67–77% of the reads were categorized as mapped reads to the porcine reference sequence.

The FPKM values were used to establish the total number of genes expressed in muscle transcriptome (>0.5 FPKM). Approximately 50% of total porcine annotated genes in the Sscrofa10.2 genome assembly were expressed in the studied samples (an average of 11,392 genes out of 22,861 annotated genes).

Ninety-five genes were overexpressed in IB (FC ranging from 1.9 to 12) and 54 genes were overexpressed in IBxDU (FC ranging from 2 to 27.2) (*p* < 0.01)(S2 Table).

Large expression differences were observed for the genes *MARCO* (27.2x) and *CXCL13* (27.1x), which showed greater expression level in IBxDU than in IB piglets. *MARCO* and *CXCL13* genes are both related to immune response. *MARCO* is also involved in cytoskeleton and cell morphology determination of certain immune cells [58] and *CXCL13*has also been found to be upregulated in adipocytes when compared to preadipocytes [59].

On the other hand, another unidentified protein (ENSSSCG00000023287; FC = 12.5x), ortholog of human *MYL6B* gene, and the pig genes *CIART* (8.4x) and *ATF3* (7.8x) were upregulated in IB piglets at birth. *CIART* is a transcription repressor of the mammalian circadian clock that inhibits the activators *CLOCK* and *BMAL-1* [60]. The mammalian circadian clock regulates sleep–wake rhythms, body temperature, blood pressure, hormone production, immune system or cell cycle (reviewed in [61]). It is also important for energy homeostasis regulation, as multiple genes involved in nutrient metabolism and metabolically related hormones such as insulin or leptin display rhythmic oscillations [62]. It has been reported that animals deficient in *BMAL-1* show altered lipid homeostasis (i.e. an increase in the levels of circulating fatty acids, including triglycerides, free fatty acids, and cholesterol) and metabolic syndrome [63], which is in agreement with the phenotypic results observed in IB pigs. *ATF3* codes for a transcription factor considered as an adaptation-response gene involved in a variety of processes such as immunity, regulation of the cell cycle and apoptosis [64], and cellular stress response [65].

Some other interesting DE genes are related to muscle and adipose tissue development, for example *SLC2A4*, *FGF21*, *UBC*, or *ACHE* [66–69]. Moreover, three DE genes (*DLK1*, *MYH10* and *ZWILCH*) were also observed to be DE between IB and IBxDU in a previous study [14], where *Longissimus dorsi* transcriptome was compared at weaning. *DLK1* is a transmembrane protein expressed in preadipocytes but not in mature adipocytes [70], thus, greater expression in IB neonates (2.6x) suggests greater number of undifferentiated preadipocytes in IB than in IBxDU piglets, possibly associated with a greater adipogenic potential. However, in a previous study, *DLK1* gene was found to be upregulated in IBxDU pigs at weaning [14]. The different age of sampling could explain the opposite results: IB piglets may be born with higher amounts of preadipocytes that differentiate faster than those from IBxDU pigs thus, leading to lower preadipocyte content at weaning age. Similarly, a different pattern for myocyte differentiation has been reported between high and low muscle growth efficiency breeds, such as Landrace, Lantang, Pietrain or Duroc [29, 71]. In Landrace, myocyte differentiation develops faster than in low efficiency breeds [29, 71]. In addition, the proliferation and differentiation of preadipocytes are stronger and faster in Bamei than in Landrace (representing a fat- and lean-type pig breed, respectively) [17, 72]. Thus, we suggest a faster adipocyte differentiation in IB pigs at an early age that may conclude earlier than in IBxDU pigs.

*MYH10* gene codes for a heavy-chain myosin, and was also found upregulated in IBxDU pigs when compared to IB pigs at birth and at weaning [14], which suggests a greater development of muscular cells in crossbreds. The gene *ZWILCH* is overexpressed in IB at both ages; it is involved in cell proliferation and differentiation, and it may also play a role in the control of adipogenesis [73], thus being an interesting candidate to explain phenotypic differences in adipogenesis and lipogenesis.

In order to validate the results obtained from the RNA-Seq analysis, the relative expression of some DE genes (upregulated in both genetic types) and a few non-DE genes was assessed by qPCR in all the available samples. Five out of the seven genes identified as DE by RNA-Seq technology were validated by qPCR. The remaining two genes showed a trend (*p* = 0.085) and a numerical difference (*p* = 0.105). Non-DE genes showed similar results using both technologies (S3 Table). A concordance correlation coefficient, used to asses technical validation in high throughput transcriptomic studies [43] was calculated (CCC = 0.93) and denoted a high general concordance between RNA-Seq and qPCR expression values. In general good individual correlation values were obtained (S3 Table). Fold-Change and significance tended to be greater when expression differences were analyzed by RNA-Seq technology, in accordance with its higher sensitivity [74].

### Biological interpretation of the differential expression results

**Fig 1:**
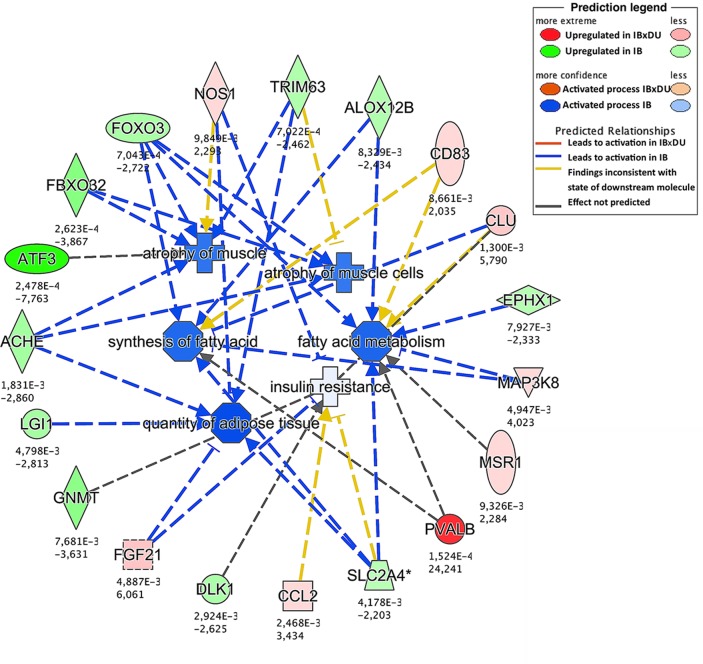
Enriched biological functions in Iberian (IB) pigs. The network generated by IPA software shows enriched biological functions in IB pigs (blue color) related to lipid metabolism and muscle atrophy. Lipid synthesis is upregulated in IB pigs. Muscle and protein degradation are also enriched, probably leading to a decreased muscle growth in those pigs. The network includes several transcription factors which may play key roles in the functional differences between genetic types, in agreement with the regulators study (*FOXO3*, *ATF3*, *TRIM63*).

**Fig 2:**
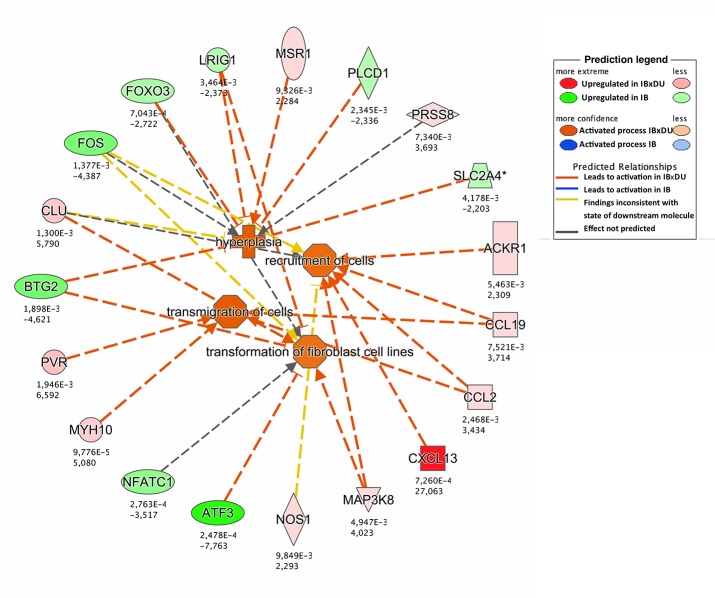
Enriched biological functions related to cell growth in crossbred (IBxDU) pigs. The network generated by IPA software shows enriched biological functions in IBxDU pigs (orange color) involved in cellular growth and differentiation. Upregulation of several chemokines as well as a myosin in IBxDU pigs, and downregulation of some transcription factors (*FOXO3*, *ATF3*) are responsible for the enrichment of such functions in crossbreds.

**Fig 3:**
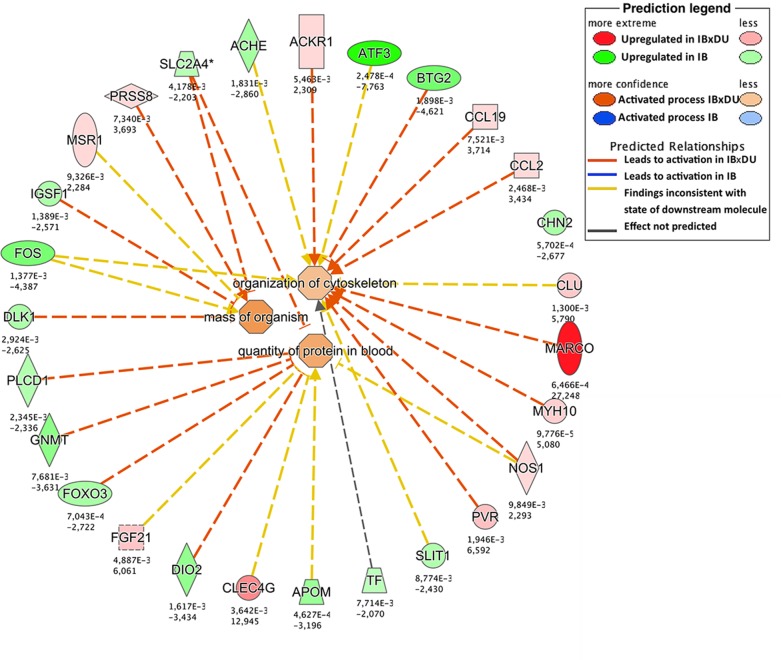
Enriched biological functions potentially related to muscle growth in crossbred (IBxDU) pigs. The network generated by IPA software shows enriched biological functions in IBxDU pigs (orange color) potentially related to muscle growth and body size, functions regulated by a wide variety of genes, from growth factors (*FGF21*) to immune system related genes (*MARCO*, *MSR1*).

**Fig 4:**
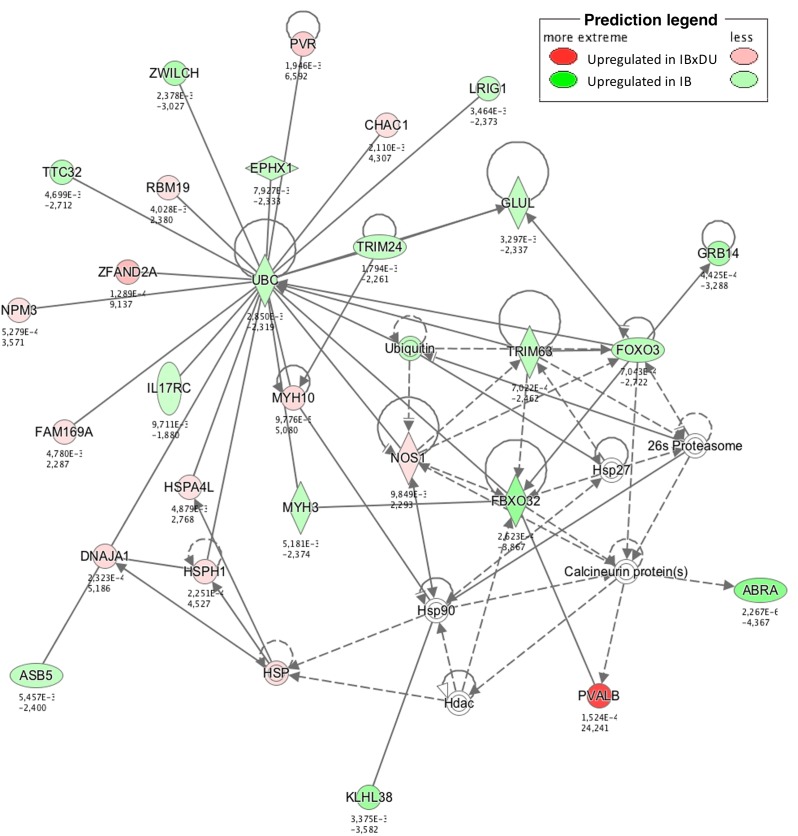
Gene network containing DE genes related to Cellular Compromise, Organismal Injury and Abnormalities and Skeletal and Muscular Disorders. This gene network shows several genes functionally interconnected and closely associated with the ubiquitin C (UBC), a central molecule in the network responsible for protein degradation. These genes are differentially expressed between genotypes and indicate the importance of the ubiquitination process in muscle growth of newborn pigs.

Different approaches were used to perform an exhaustive and robust biological interpretation of the results obtained in the transcriptome study. Results obtained from the Gene Ontology (GO) term overrepresentation analysis, performed to detect active biological processes differing in both IB and IBxDU, are shown in Table 2. In addition, IPA software was used to find biological functions overrepresented in both genetic types (S4 Table and Figs 1–3) and to identify pathways (Table 3) and networks (Fig 4) associated with the DE genes.

**Table 2:** Gene Ontology (GO) overrepresented terms regarding the biological process category.

	*GO term	*p-*value	*q*-value	Term name
COMMON	GO:0007165	9.54E-07	6.96E-05	Signal transduction
	GO:0031323	3.81E-06	1.39E-04	Regulation of cellular metabolic process
	GO:0009059	6.10E-05	6.37E-04	Macromolecule biosynthetic process
	GO:0044267	1.22E-04	8.10E-04	Cellular protein metabolic process
	GO:0010646	2.44E-04	1.11E-03	Regulation of cell communication
	GO:0030154	2.44E-04	1.11E-03	Cell differentiation
	GO:0048584	7.81E-03	1.84E-02	Positive regulation of response to stimulus
IB^1^	GO:0010467	1.22E-04	8.10E-04	Gene expression
	GO:0043412	1.22E-04	8.10E-04	Macromolecule modification
	GO:0036211	2.44E-04	1.11E-03	Protein modification process
	GO:0009889	4.88E-04	1.98E-03	Regulation of biosynthetic process
	GO:0090304	9.77E-04	3.56E-03	Nucleic acid metabolic process
	GO:0019438	1.95E-03	5.48E-03	Aromatic compound biosynthetic process
	GO:0016070	1.95E-03	5.48E-03	RNA metabolic process
	GO:0023056	7.81E-03	1.84E-02	Positive regulation of signaling
IBxDU^2^	GO:0002684	1.95E-03	1.86E-02	Positive regulation of immune system process
	GO:0050790	7.81E-03	3.49E-02	Regulation of catalytic activity
	GO:0016337	7.81E-03	3.49E-02	Single organismal cell-cell adhesion

^1^ IB = Purebred Iberian pigs

^2^ IBxDU = Iberian X Duroc crossbred pigs

*GO terms are considered either common, when they are enriched in both genetic types and specific when the GO term is only enriched in one of the two genetic types.

**Table 3:** Pathways significantly enriched in Purebred (IB) and Duroc-crossbred (IBxDU) Iberian pigs.

IB^1^		IBxDU^2^	
Pathway	*p-*value	Pathway	*p-*value
PI3K Signaling in B Lymphocytes	<0.001	Agranulocyte Adhesion and Diapedesis	<0.001
NRF2-mediated Oxidative Stress Response	0.0022	LXR/RXR Activation	<0.001
Glutamine Biosynthesis I	0.0031	Atherosclerosis Signaling	<0.001
IL-17A Signaling in Fibroblasts	0.0051	Aldosterone Signaling in Epithelial Cells	0.0012
April Mediated Signaling	0.0060	Granulocyte Adhesion and Diapedesis	0.0019
LXR/RXR Activation	0.0060	TREM1 Signaling	0.0050
Epoxysqualene Biosynthesis	0.0062	Protein Ubiquitination Pathway	0.0054
B Cell Activating Factor Signaling	0.0066	Citrulline-Nitric Oxide Cycle	0.0071
Thyronamine and Iodothyronamine Metabolism	0.0091	IL-12 Signaling and Production in Macrophages	0.0155
Thyroid Hormone Metabolism I (via Deiodination)	0.0091	Superpathway of Citrulline Metabolism	0.0195
Wnt/Ca+ pathway	0.0129	nNOS Signaling in Skeletal Muscle Cells	0.0209
Tight Junction Signaling	0.0145	Differential Regulation of Cytokine Production in Macrophages and T Helper Cells by IL-17A and IL-17F	0.0251
AcutePhase Response Signaling	0.0151	Production of Nitric Oxide and Reactive Oxygen Species in Macrophages	0.0263
ERK5 Signaling	0.0158	Clathrin-mediated Endocytosis Signaling	0.0275
Growth Hormone Signaling	0.0191	Actin Cytoskeleton Signaling	0.0372
Clathrin-mediated Endocytosis Signaling	0.0191	Role of p14/p19ARF in Tumor Suppression	0.0417
Melatonin Signaling	0.0195	IL-17A Signaling in Fibroblasts	0.0468
Toll-like Receptor Signaling	0.0214		
Regulation of IL-2 Expression in Activated and Anergic T Lymphocytes	0.0245		
UVA-Induced MAPK Signaling	0.0295		
Antioxidant Action of Vitamin C	0.0355		
IGF-1 Signaling	0.0355		
T Cell Receptor Signaling	0.0355		
Role of IL-17A in Psoriasis	0.0389		
Cholesterol Biosynthesis I	0.0389		
Cholesterol Biosynthesis II (24. 25-dihydrolanosterol)	0.0389		
Cholesterol Biosynthesis III (via Desmosterol)	0.0389		
Vitamin-C Transport	0.0417		
Glucocorticoid Receptor Signaling	0.0457		
FattyAcid α-oxidation	0.0479		

^1^ IB = Purebred Iberian pigs

^2^ IBxDU = Iberian X Duroc crossbred pigs

#### Upregulated functions and pathways in *Biceps femoris* muscle from purebred Iberian piglets

Enriched biological functions in IB piglets identified by IPA software were mainly related with lipid and glucose metabolism (i.e. *Concentration of lipid*, *Synthesis of lipid*, *Insulin resistance*, *Quantity of adipose tissue* or *Fatty acid metabolism;* Fig 1) and with muscle growth (S4 Table). In agreement, several DE genes with known functions related to lipid metabolism such as *FOS*, *FDFT1*, *SLC4A2*, *TRIM63*, *EPXH1*, *ALOX12B*, *FOXO3A* or *ACHE* were overexpressed in IB, possibly associated to the higher amount of adipose tissue observed in BF muscle of IB when compared to IBxDU pigs (Table 1). Similar results have been found in IB and IBxDU piglets at 28 days of age in loin muscle [14]. However, in the previous study, a different set of DE genes related to lipid metabolism was found, probably due to the high complexity of processes and molecular mechanisms regulating lipid metabolism at that stage of development. In another study comparing the muscle transcriptome of two divergent breeds for muscle and fat deposition, several muscle metabolism-related genes were identified as potential regulators of IMF deposition [29], such as *FOS* and *ABRA* genes, identified also in the present study.

Accordingly, several pathways enriched in IB pigs (*p* < 0.05) (Table 3) were related to the control of energy homeostasis (*Wnt/Ca+*, *Glutamine Biosynthesis* or *Fatty Acid α-oxidation* pathways)and to protein synthesis and cell growth (*Growth Hormone* (*GH) Signaling* and *IGF-1 Signaling*); IGF-1 is essential during prenatal and GH during postnatal growth [75]. The effect of GH and IGF-1 on adipose tissue development and metabolism is controversial, as both have been proposed to play either a positive or a negative role on adipocyte differentiation [76, 77]. Thus, in IB newborns, the activation of these pathways might be associated to both muscle and preadipocytes development and differentiation. Accordingly, the *adipogenesis pathway* showed a trend for enrichment (*p* = 0.055).

#### Upregulated functions and pathways in *Biceps femoris* muscle from Duroc-crossbred Iberian piglets

Enriched functions in IBxDU newborns (S4 Table) were related to cell growth and differentiation, such as *Recruitment and transmigration of cells*, *Hyperplasia or Transformation of fibroblast cell lines* (Fig 2), and to protein and muscle organization and accretion such as *Mass of organism*, *Quantity of protein in blood* and *Organization of cytoskeleton* (Fig 3). Consistently, DE genes in IBxDU pigs clustered in 3 gene networks related to *Cell-To-Cell Signaling and Interaction*, *Hematological System Development and Function*, *Immune Cell Trafficking*, *Post-Translational Modification*, *Protein Folding*, *Cellular Assembly and Organization*, *Cellular Movement*, *Cell Signaling* and *Cellular Function and Maintenance*.

Some IBxDU upregulated genes involved in those functions were chemokines (i.e.*CCL19*, *CCL2* or *CXCL13*) and chemokine receptors (*ACKR1*), which have been reported to be involved in the process of myogenesis and muscle regeneration [78]. In our study, chemokines were associated with functions such as transmigration and recruitment of cells and organization of cytoskeleton (Figs 2 and 3), which could be related to muscle cell growth. Moreover, *MYH10*, a non-muscle myosin that regulates actin cytoskeleton remodeling, was found overexpressed in IBxDU and involved in cytoskeleton reorganization, a critical process during myogenesis [79]. Consistently, the *Actin Cytoskeleton Signaling* pathway was also enriched in IBxDU pigs in both the present study and at 28 day of age [14].

*Mass of organism*, associated with the overexpression of *PRSS8*, was predicted by IPA software to be enriched in IBxDU piglets, in accordance with the phenotypic differences in body weight and size found between pure and crossbred pigs. In agreement, a decrease in body weight was reported in mutant rats for gene *PRSS8* [80].

The enrichment of protein and muscle development-related pathways (*p* < 0.05), such as *Citrulline-Nitric Oxide Cycle*, *Superpathway of Citrulline Metabolism or nNOS Signaling in Skeletal Muscle*, support the greater muscle development in IBxDU piglets. Citrulline is a non-essential amino acid that plays a role in muscle development and affects body composition in rats, increasing lean and decreasing adipose tissue when it is provided in the diet [81]. The *nNOS Signaling in Skeletal Muscle Cells* modifies the blood flow to the muscle [82]. Thus, the activation of these pathways suggests a greater nutrient input to muscle tissues in those pigs.

#### Upregulated functions and pathways in *Biceps femoris* muscle from both Duroc-crossbred and purebred Iberian piglets

Development-related processes depend on the genetic background, but also on the growth stage. At birth, when growth is a very active process, several functions and pathways enriched in both genetic types were observed. However, these common processes ended up in different phenotypic consequences. This might be because developmental mechanisms such as muscle growth or immune system development are tightly regulated and the final output depends on both the balance of activating and inhibiting pathways and the expression levels of genes involved in such processes.

Regarding cellular growth, the GO terms enrichment analysis retrieved several GO terms related to normal cell cycle and growth, such as cell differentiation, cellular protein metabolic process or macromolecule biosynthetic process that were common between both genetic types, supporting the importance of these processes in the early development of pigs. Accordingly, in 3 months-old Casertana pig (an autochthonous Italian fatty pig breed), GO terms close to the aforementioned were upregulated when compared to 6 months old pigs [83].

The importance of protein metabolism at birth is supported by the activation of the protein ubiquitination process in both genetic types. The *Protein Ubiquitination Pathway* was enriched in crossbred piglets (*p* = 0.005; Table 3), where mainly members of the family of Heat Shock Proteins (*HSPH1*, *HSPA4L* and *DNAJA1*) were found upregulated in IBxDU pigs. *DNAJA1*, together with *HSP27* have been reported to play a role in both cellular stress and meat quality; their expression was found to decrease IMF content [84], consistently with the results observed in IBxDU pigs. However, no enrichment in atrophy or degradation of muscle-related functions was observed in IBxDU piglets. Conversely, IB muscle showed activation of functions related to muscle atrophy (Fig 1), mainly driven by genes that stimulate muscle atrophy by means of the ubiquitin proteasome system (UPS) [85]. This system was also upregulated in IB when compared to IBxDU pigs at 28 days of age [14]; and in Basque (a French pig breed with low lean meat and high fat contents) when compared to Large White pigs [86]. Supporting the evidence of an activated protein ubiquitination process, the gene coding for Ubiquitin C (*UBC*) and *FBXO32* (one of the three ligase enzymes, together with *TRIM63* of the UPS) were also overexpressed in IB pigs.

In accordance with these results, genes implicated in these processes were found in the most significant gene network involving DE genes in both genetic types. This network was associated with *Skeletal and Muscular Disorders*, as well as with *Cellular Compromise*, *Organismal Injury and Abnormalities* (Fig 4) and showed several genes related to protein catabolism (*UBC*, *HSP*, *TRIM63* or *FOXO3*) among the most central genes in the network, which supports the importance of these genes in protein catabolism, a very active process in developing IBxDU and especially in IB pigs.

Iberian pigs show low protein accretion potential, although higher relative protein synthesis rate has been reported in IB than in lean Landrace pigs [87]. Since IB muscles are smaller than Landrace ones, it was suggested that a higher protein turnover rate in IB pigs should be responsible for the differences between protein synthesis and deposition [87]. In this context, it seems evident that this increased protein turnover in IB pigs is related to the activated muscle atrophy-related genes found in this study. Moreover, Damon et al. (2012) suggested that the activation of the UPS might affect meat tenderness, since proteasome would be one of the main endogenous proteolityc systems contributing post-mortem meat tenderization.

The UPS is also interconnected to the unfolded protein response (UPR), activated under endoplasmic reticulum stress situations. The UPR is involved in degradation of un and misfolded proteins and recently associated to the control of adipogenesis under situations of endoplasmic reticulum stress [88, 89]]. It is noteworthy that *ATF3*, a transcription factor inducible by endoplasmic reticulum stress [65] was upregulated in IB pigs. This could suggest certain level of endoplasmic reticulum stress in IB pigs that might be related to preadipocyte differentiation.

**Fig 5:**
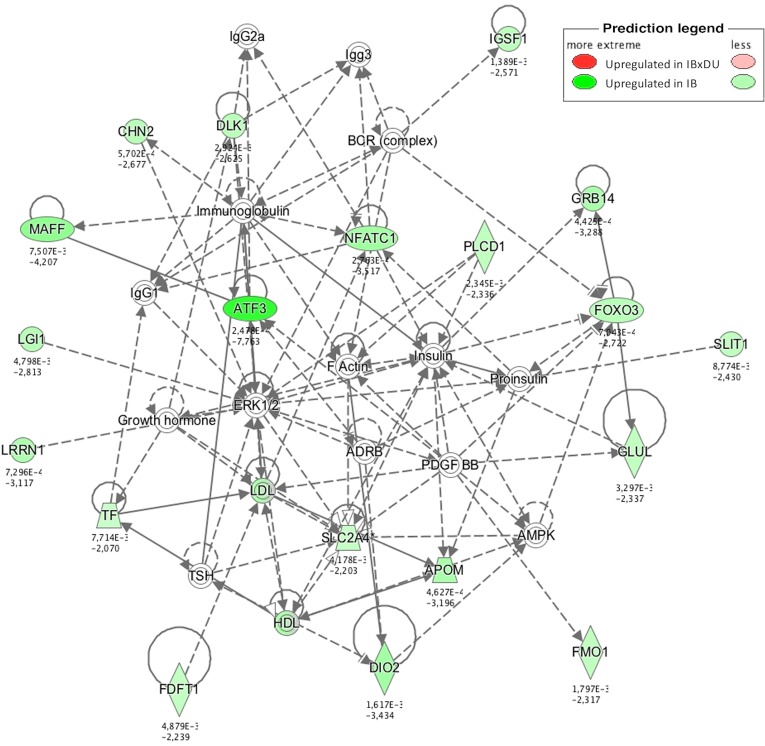
Gene network containing genes upregulated in Iberian (IB) pigs related to *Amino Acid Metabolism*, *Molecular Transport* and *Small Molecule Biochemistry*. The gene network shows interactions between cholesterol and lipid metabolism-related genes (LDL, APOM, FDFT1, SLC2A4, etc.) upregulated in Iberian pigs.

The importance of cholesterol in early stages of development could be the cause for the activation of pathways related to cholesterol metabolism and biosynthesis in both genetic types. For example, the *LXR/RXR activation pathway*, involved in the regulation of lipid metabolism, inflammation and cholesterol to bile acid catabolism, was enriched in both IB and IBxDU newborns (*p* = 0.006 and *p* < 0.001, respectively). On the contrary, at 28 days of age, this pathway was enriched in IB pigs [14]. The *atherosclerosis signaling pathway* (enriched in IBxDU pigs) or the *Cholesterol Biosynthesis* and the *Epoxysqualene Biosynthesis pathways* (enriched in IB pigs) were also identified as relevant pathways. However, phenotypic observations reflect a more active cholesterol and triglycerides biosynthesis in IB than in IBxDU piglets (Table 1).This could be due to the upregulation of different genes in each genetic type: upregulated genes in IB pigs associated with these pathways were closely related to the cholesterol and lipid metabolism (*FDFT*, *TF* and *APOM*). Accordingly, genes related to cholesterol and lipid metabolism such as *RXRG*, *USF1*, *LPL* or some apolipoproteins have been proposed as candidate genes involved in the familial combined hyperlipidemia in human [56, 90, 91], characterized by high levels of plasma cholesterol and triglycerides, as observed in IB pigs. Moreover, the aforementioned upregulated genes in IB pigs were connected in a network associated with functions such as *Amino Acid Metabolism*, *Molecular Transport* and *Small Molecule Biochemistry* (Fig 5). On the other hand, genes involved in such cholesterol-related pathways that were upregulated in IBxDU piglets where associated to the immune response (*MSR1* and *CCL2*).

The immune system is not fully developed at birth in pigs [92], and thus, related pathways and functions were upregulated in both in IB and IBxDU newborns. Some of these pathways include *PI3K Signaling in B Lymphocytes*, *April Mediated Signaling* or *B Cell Activating Factor Signaling* (enriched in IB pigs) and *Agranulocyte Adhesion and Diapedesis* or *IL-12 Signaling and Production in Macrophages* (enriched in IBxDU pigs). Moreover, the pathway *IL-17A Signaling in Fibroblasts* was enriched in both genetic types. On the other hand, the GO term *Positive regulation of immune system process* was enriched in IBxDU but not in IB piglets. This is consistent with the aforementioned upregulation of genes such as *MARCO* and *CXCL13* in Duroc-crossbred pigs, suggesting a more developed immune system in IBxDU than IB newborns. It has been previously reported that the immune system is affected by pig breed [93] and domestication [94]. An overrepresentation of genes related to the immune system was reported in wild boars when compared to domestic pig breeds [95]. However, to our knowledge there are no previous studies assessing differences in immune efficiency introduced by sire line.

Immune system-related genes are also involved in numerous other biological processes, such as fat accumulation, closely associated with inflammation [96, 97]. Thus, the enrichment of pathways related to the immune system could be related to lipid accumulation, although it has also been related to lower backfat thickness in pigs [98]. It is noteworthy that no biological functions related to the immune system were found enriched in IB pigs, which might suggest that upregulation of immune system-related pathways might be related to adipose tissue development rather than to the immune system development in IB pigs.

The reported active biological functions in IB and IBxDU pigs suggest a different predisposition for cell and tissue growth between genetic types, being IB pig metabolism intended to energy storage and IBxDU pig metabolism to cell growth and differentiation. These differences in biological regulation are in agreement with phenotypic differences found between the two genetic types.

### Regulatory transcription factors

We performed a regulatory factors study to investigate the driving molecular mechanisms responsible for the differences in gene expression observed between genetic types.

A total of 723 TRF, obtained from the animal TFDB, showed expression values above 0.5 FPKM in our pigs and were thus considered as expressing TRF. Among them, 92 TRF potentially affected the gene expression profile in IB and IBxDU muscles (Table 4). We considered TRF that were either DE (7 TRF), identified by IPA software as regulators (45 TRF) or identified in the RIFs study (48 TRF) (Table 4).

**Table 4:** Potential regulators affecting gene expression that are: a) differentially expressed (DE) between IB and IBxDU; b) identified by Ingenuity Pathways Analysis (IPA) software or c) identified by RIFs study.

Ensembl ID	GENE	DE regulators		IPA—Regulators		RIFs study	
		*p-*value	FC Change	Z-score	*p-*value	RIF1(z)	RIF2(z)
ENSSSCG00000006036	*ABRA*	2.27E-06	-4.37				
ENSSSCG00000008123	*ARID5A*						2.01
ENSSSCG00000015972	*ATF2*				4.40E-02		
ENSSSCG00000015595	*ATF3*	2.48E-04	-7.76				
ENSSSCG00000000084	*ATF4*				1.42E-02		
ENSSSCG00000012241	*BCOR*				4.17E-03		
ENSSSCG00000013397	*BMAL1*						-2.22
ENSSSCG00000008377	*CCT4*						-2.11
ENSSSCG00000002866	*CEBPA*			-2.426	7.32E-03		
ENSSSCG00000002867	*CEBPG*					3.60	
ENSSSCG00000006752	*CSDE1*					2.45	
ENSSSCG00000011274	*CTNNB1*			-2.183	1.01E-02		
ENSSSCG00000014336	*EGR1*			-0.254	5.28E-03		
ENSSSCG00000010224	*EGR2*				2.01E-02		2.21
ENSSSCG00000008443	*EPAS1*				4.01E-02		
ENSSSCG00000002383	*FOS*	1.38E-03	-4.39	0.381	6.14E-03		
ENSSSCG00000012967	*FOSL1*				2.76E-02		
ENSSSCG00000007576	*FOXK1*					2.35	
ENSSSCG00000009370	*FOXO1*				1.03E-02	3.22	
ENSSSCG00000004387	*FOXO3*	7.04E-04	-2.72	-2.364	5.05E-06		
ENSSSCG00000001619	*FOXP4*					2.79	
ENSSSCG00000015733	*GLI2*					2.60	
ENSSSCG00000007720	*GTF2IRD1*				1.66E-02		
ENSSSCG00000000846	*HCFC2*					2.34	
ENSSSCG00000014388	*HDAC3*				2.36E-02		
ENSSSCG00000010472	*HHEX*				4.89E-02		
ENSSSCG00000004138	*HIVEP2*				1.70E-04		
ENSSSCG00000009327	*HMGB1*				3.67E-03		
ENSSSCG00000009704	*HMGB2*					2.55	
ENSSSCG00000008898	*HOPX*				1.66E-02		
ENSSSCG00000015985	*HOXD3*					3.59	
ENSSSCG00000005917	*HSF1*				9.28E-05		
ENSSSCG00000004238	*HSF2*				6.06E-03		
ENSSSCG00000014277	*IRF1*				2.49E-02		2.19
ENSSSCG00000003178	*IRF3*				3.13E-02		
ENSSSCG00000012853	*IRF7*				3.08E-02		
ENSSSCG00000008119	*KCNIP3*					2.34	
ENSSSCG00000010928	*KDM5B*						-2.10
ENSSSCG00000006928	*LMO4*					2.64	
ENSSSCG00000004528	*MBD2*				2.44E-02		
ENSSSCG00000000552	*MED21*					2.35	
ENSSSCG00000005720	*MED27*					2.18	
ENSSSCG00000006482	*MEF2D*				6.93E-03		
ENSSSCG00000012534	*MORF4L2*						-2.11
ENSSSCG00000013507	*MPND*						2.18
ENSSSCG00000012114	*MSL3*						2.34
ENSSSCG00000017882	*MYBBP1A*				2.07E-02		
ENSSSCG00000013375	*MYOD1*				7.26E-03		
ENSSSCG00000015475	*MYOG*				8.17E-06		
ENSSSCG00000010399	*NCOA4*					3.59	
ENSSSCG00000015987	*NFE2L2*			-2.206	2.44E-02		
Ensembl ID	GEN	DE regulators		IPA—Regulators		RIFs study	
		*p-*value	FC	Z-score	*p-*value	RIF1(z)	RIF2(z)
ENSSSCG00000001952	*NFKBIA*			-1.279	3.40E-02		
ENSSSCG00000001703	*NFKBIE*				4.09E-02		
ENSSSCG00000005385	*NOR-1*	8.17E-04	-6.56				
ENSSSCG00000009856	*NOS1*	9.85E-03	2.29		3.78E-05		
ENSSSCG00000006689	*PIAS3*				3.69E-02		
ENSSSCG00000014437	*PPARGC1B*				1.65E-02		
ENSSSCG00000009746	*RAN*						-2.25
ENSSSCG00000009401	*RB1*			1.172	2.29E-02		
ENSSSCG00000008388	*REL*				2.04E-03		
ENSSSCG00000012981	*RELA*			0.749	6.16E-04		
ENSSSCG00000017071	*SAP30L*						-2.28
ENSSSCG00000011201	*SATB1*						-2.32
ENSSSCG00000001880	*SIN3A*						-2.12
ENSSSCG00000004952	*SMAD2*						2.43
ENSSSCG00000004524	*SMAD4*				4.94E-02		
ENSSSCG00000005232	*SMARCA2*						-2.47
ENSSSCG00000013629	*SMARCA4*				4.00E-02		
ENSSSCG00000006256	*SOX17*				3.29E-02		
ENSSSCG00000017403	*STAT3*				3.52E-02		
ENSSSCG00000017406	*STAT5B*					2.22	
ENSSSCG00000010638	*TCF7L2*				2.68E-02		
ENSSSCG00000001092	*TDP2*					2.44	
ENSSSCG00000001544	*TEAD3*						-2.06
ENSSSCG00000017950	*TP53*			-1.444	1.21E-04	3.48	1.69
ENSSSCG00000007208	*TRIB3*					2.84	
ENSSSCG00000027684	*TRIM63*	7.02E-04	-2.46		1.25E-02		
ENSSSCG00000015370	*TWIST1*				3.28E-03		
ENSSSCG00000006790	*WDR77*						-2.18
ENSSSCG00000011953	*ZBTB11*					2.10	
ENSSSCG00000007958	*ZNF174*					2.31	
ENSSSCG00000003233	*ZNF175*					2.86	
ENSSSCG00000002838	*ZNF423*					2.26	
ENSSSCG00000003070	*ZNF45*					2.24	
ENSSSCG00000007767	*ZNF668*						2.26
ENSSSCG00000001206	*ZSCAN26*						-2.84
ENSSSCG00000002877	*ZNF181*					2.50	
ENSSSCG00000003244							2.05
ENSSSCG00000011620							-2.41
ENSSSCG00000011943							-2.07
ENSSSCG00000013384							1.96
ENSSSCG00000016700						3.43	

FC: Fold-Change

Z-score: reflects the activation state of predicted transcriptional regulators. It is based on the experimentally observed gene expression, and on literature‐derived regulation direction information, which can be either “activating” or “inhibiting”

RIF1 (z) extreme scores identify those transcription factors that are consistently most differentially co-expressed with highly abundant and highly DE genes. Bootstrap 95% and 99% confidence intervals for RIF1 z-scores: -1.996/2.074 and -2.883/2.918, respectively.

RIF2 (z) extreme scores identify transcription factors with the most altered ability to predict the abundance of DE genes. Bootstrap 95% and 99% confidence intervals for RIF2 z-scores: -2.036/1.953 and -2.609/2.490, respectively

In the present study, we found 7 TRF showing differences in expression level between genetic types. Most of them (*ABRA*, *ATF3*, *FOS*, *FOXO3*, *NOR1*, *TRIM63*) were upregulated in IB. These genes are related to muscle and adipose tissue development (Fig 1) and most of them have been mentioned in the previous section, but some of them may be highlighted and deserve additional comments. For example, one of the genes with greater expression differences (*ATF3*) was also an identified regulator in the *Animal TFDB*, suggesting an important role in the gene expression differences observed. *NOR1* showed also an important expression difference (6.56x) and codes for a nuclear receptor involved in a wide array of functions such as inflammation, cell cycle regulation, apoptosis, steroidogenesis, adipogenesis, angiogenesis and energy metabolism. Moreover, it has been found overexpressed in obese when compared to normal humans and its expression went back to normal values after fat loss [99]. These findings are in accordance with the results of the present study, where animals with higher IMF content showed greater muscular expression of *NOR1*.

IPA software is a potent tool to identify regulators based on previous knowledge and bibliographic references. On the other hand, the RIFs analysis, based on co-expression information in our dataset, complements the bibliographic approach (IPA). The combination of these two methods is a powerful strategy to identify TRF. In the present work, IPA software identified 45 TRF affecting the DE genes, while the RIFs metrics identified 48. Four TRFs were identified following the two approaches (*EGR2*, *FOXO1*, *IRF1* and *TP53*). *EGR2* is a growth factor that promotes adipocyte differentiation [100], while *TP53* has been reported to affect cell metabolism [101, 102]. *FOXO1* is a member of the forkhead family of TRF, which exerts important regulatory functions in developmental processes including muscle development [103, 104]. *FOXO1* regulates expression of several adipogenic genes, including *PPARG* [105] and muscle cells differentiation in association with *SMAD* [106], although inconsistent information exits regarding its specific role [106, 107]. Moreover, several TRF with known functions on adipogenesis and lipid metabolism were identified in the present regulators analysis (*CEBPA*, *CEBPG*, *ZFP423*, *EGR1*, *ATF2*, *ATF4* and *PPARGC1B)*. *PPARGC1B*is involved in fat oxidation, non-oxidative glucose metabolism, mitochondrial biogenesis and the regulation of the energy expenditure [108] and was also identified as a potential regulator of gene expression differences at 28 days of age [14]. The identification of these genes as regulators in the present study suggests its implications in adipogenesis and in the observed fatness differences associated to sire breed.

Regulation of myogenesis is also a very complex process and several TRF involved on it have been identified (*FOXK1*, *FOXO4*, *MEF2D*, *MYOD1*, *HDAC3* and *MYOG)*. These genes play a central role in myogenesis regulation, acting sequentially in a signaling chain [29, 109–113]. Due to the importance of muscle development in the pork industry, several studies have assessed the differential expression of myogenic regulatory factors between breeds with different muscle growth potential [20, 29, 83, 114]. The identification of these myogenic regulators as TRF in the present study, suggests that myogenesis is also activated immediately after birth, although it has been reported that expression significantly decreases in pigs after 90 days post-conception [114]. However, muscle development timing remains unclear, since it has also been hypothesized that muscle is still under development in 3 months-old Casertana pigs [83]. Moreover, genes involved in myogenesis inhibition (i.e. *SMAD* and *TWIST1*) were also identified as TRF affecting gene expression in IB and IBxDU pigs. The presence of both myogenesis activating and inhibiting genes reflects the complex regulation of this process in newborn piglets.

Muscle deposition not only depends on myogenesis; regulation of process such as angiogenesis, or protein degradation is also decisive in final muscle deposition. SOX17 and the heat shock transcription factor family members HSF1 and HSF2, involved in such processes [115–117], were identified in this TRF study.

**Fig 6:**
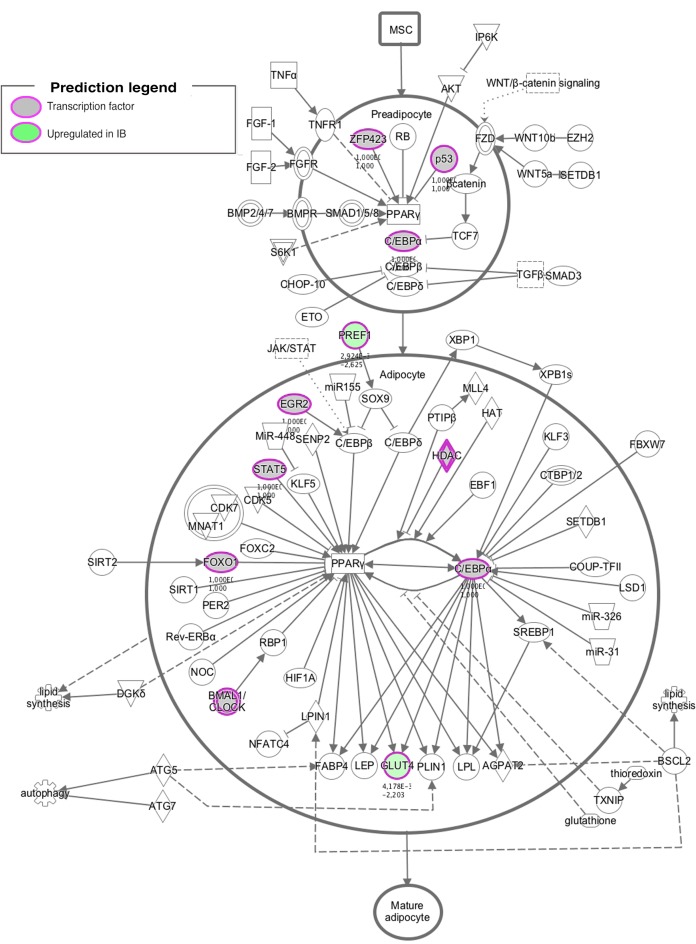
Adipogenesis pathway. Several transcription factors identified in the regulators study and two genes upregulated in Iberian pigs (*PREF1* and *GLUT4*) are involved in the adipogenesis pathway. Most of them are involved in *PPARG* expression or activation.

To better understand interactions between the identified DE genes and TRF, a pathways enrichment analysis was performed combining information obtained in both analyses. The adipogenesis pathway was the most enriched pathway (S5 Table), accordingly with the differences in fatness observed in *BF* muscle of IB and IBxDU piglets. As observed in Fig 6, the adipogenesis pathway includes several TRF (*ZFP423*, *P53*, *FOXO1*, *STAT5* or *EGR2*) and DE genes (*SLC4A2/GLUT4* and *DLK1*). It is noteworthy that *PPARG*, considered as the master regulator of adipogenesis, was not found to be DE or to regulate gene expression in newborn IB and IBxDU pigs. However, 6 out of the 8 identified TRFs involved in this pathway, regulated directly or indirectly the expression of *PPARG*.

### Structural analysis from RNA-Seq sequence data

A total of 433,667 putative variants were identified in IB and 461,438 in IBxDU pigs.

The total numbers of polymorphisms meeting the filtering criteria, and the location, frequency, aminoacid change and variant type distribution in both genetic types are shown in Table 5. Both genetic types showed a similar variant number, with similar location and variant type distributions. Regarding variant frequency distribution, IB group showed slightly higher number of potentially fixed variants (frequency equal or higher than 90%) while crossbreds showed higher number of segregating variants (frequency lower than 90%), in agreement with the expectations for the comparison of one pure line with high inbreeding level as the Iberian pig [23] and an F1 cross. Nevertheless, frequency estimations have to be considered with caution due to the limited sample size. Variants at different allelic frequencies in both genetic types were of special interest, especially those potentially fixed in IB and segregating in IBxDU (7,741 variants). Moreover, variations in protein-coding regions may give rise to non-synonymous changes in the amino acid sequence of the encoded protein and thus, are more likely to affect the protein structure and function [118] probably leading to critical effects on a phenotype of interest [119]. Out of the 7,741 potentially relevant variants, 846 produced a non-synonymous change.

**Table 5:** Number of sequence variants present in IB and IBxDU pigs RNA-Seq data and its distribution according to localization, frequency and polymorphism type.

	IB	IBxDU
Total number of variants (after filtering)	120,998	125,382
Variant localization		
UTR Region	20,683	21,808
Coding region	100,315	103,574
Potentially fixed and segregating variants		
Fixed (γ≥90%)	46336	40454
Segregate (γ<90%)	74662	84928
AA change	18092	18556
Variant type		
SNV	88,415	92,416
MNV	11,842	11,873
Insertion	7,863	7,987
Deletion	9,539	9,699
Replacement	3,339	3,407

IB: Iberian purebred piglets

IBxDU: Duroc crossbred Iberian piglets

Filtering: Variants with coverage < 30 or frequency < 5% were dismissed

UTR Region: Untranslated region

AA change: Variants causing a aminoacid change in the protein

SNV: Single nucleotide variant

MNV: Multiple nucleotide variant

Replacement: Neighboring SNVs and insertions or deletions.

One of the goals of the present study was to identify structural variants in candidate genes involved in differences between IB and IBxDU piglets. Candidate genes were selected based on the following criteria: TRF simultaneously identified following IPA and RIF approaches (*EGR2*, *FOS*, *FOXO1*, *FOXO3*, *IRF1*, *NOS1*, *TRIM63*, *ABRA*, *ATF3* and *TP53*) and those coinciding with TRF previously identified in an IB and IBxDU piglets transcriptome comparison at 28 days of age (*HOXA9*, *STAT5B*, *ATF4* and *PPARGC1B*).

In order to identify structural variants in these strong candidate genes, we looked for differentially segregating SNPs in IB and IBxDU pigs. The total variant number in the whole set of selected genes was slightly larger in IBxDU than in IB pigs (365 and 294, respectively). Out of the identified variants, 177 were present in both genetic types, 117 were present uniquely in IB and 188 in IBxDU piglets. The supplementary file 6 (S6 Table) shows all the variants found in the 14 genes of interest. The genes *ATF4* and *HOXA9* did not show any polymorphism. The genes *ATF3*, *STAT5* and *ABRA* showed common polymorphisms between the two genetic types that were fixed or in high allelic frequency in IB pigs. Among them, *ABRA* presented 11 variants fixed in IB that segregate in IBxDU. None of these polymorphisms led to aminoacidic changes, nevertheless they might affect mRNA processing (splicing, maturation, stability, transport) and translation and thus, lead to altered mRNA folding and even expression [120]. Moreover, the 11 variants were found in cosegregation, which might lead to relevant changes in the mRNA. The identified forkhead family TRFs (*FOXO1* and *FOXO3A*) showed fixed and high frequency variants uniquely present in IBxDU pigs. Nevertheless, we identified a low frequency variant in IB pigs that produced a non-synonymous amino acid change in *FOXO1* gene. *NOS1* and *TP53* genes presented several variants in both genetic types. Variants in *NOS1* gene were mainly associated to the IBxDU type, two of them causing amino acid changes. On the other hand, variations in *TP53* were observed generally in IBxDU pigs. Considered as a tumor suppression gene, TP53 protein responds to diverse cellular stresses to regulate expression of target genes, thereby inducing cell cycle arrest, apoptosis, senescence, DNA repair, or changes in metabolism. It has been recently reported as a novel candidate for muscle development in pigs [121] and was also involved in the adipogenesis pathway (Fig 6). The *PPARGC1B* and *TRIM63* genes were the most polymorphic ones among the studied genes (89 and 94 variants, respectively). Some of the variants found in these genes were associated to amino acid change. One of the variants identified in the *PPARGC1B* gene, at medium frequency in IB and absent in IBxDU, was predicted by SIFT to produce a deleterious amino acid change. Also a GCA-deletion, fixed in IB and segregating in IBxDU pigs, produces the lack of an alanine residue in the coded PPARGC1B protein. On the other hand, 15 variants raised missense amino acid changes and 7 produced frameshift alteration in the *TRIM63* gene, which maintains muscle protein homeostasis by tagging the sarcomere proteins with ubiquitin for subsequent degradation by the UPS [122]. Moreover the polymorphism *ENSSSCT00000028044*:*c*.*163G>C* was predicted to be deleterious by SIFT metrics. Thus, these changes might potentially cause altered function in the proteins. Previous studies associated polymorphisms in *PPARGC1B* gene with type 2 diabetes [123] and subcutaneous adiposity [124] in human. Moreover, *PPARGC1A* codes for a homologous protein of *PPARGC1B* and has been associated in pigs to carcass composition traits such as leaf fat weight, backfat thickness, and belly weight in a Meishan cross population [125].

Variants showing different segregation between genetic types may be associated with functional genetic differences, which potentially could affect gene regulation and metabolism. Specially, the polymorphisms associated to aminoacid changes detected in *FOXO1*, *NOR-1* and *PPARGC1B* genes might be candidate polymorphisms to partially explain the differential muscle and adipose growth between IB and IBxDU.

## Conclusions

In the present study, differences in phenotype, transcriptome, metabolic pathways and transcriptional regulation were found between BF muscles from purebred and Duroc-crossbred Iberian newborn pigs. Phenotypic differences regarding body size, plasma cholesterol levels and IMF content were remarkable, even at this early age. This is concordant with the transcriptomic study, which revealed several DE genes related to adipose and muscle tissues development such as *DLK1*, *FGF21* and *UBC* genes. The interpretation of these results pointed out a differential regulation of several biological processes. For example, lipid metabolism and muscle atrophy were upregulated in IB pigs, in accordance with the greater adipose accretion and lower muscle growth observed in Iberian pig breed. However, muscle growth was upregulated in IBxDU pigs, characterized for a higher muscle development than IB pigs. These processes are closely related to meat quality and production traits. Protein catabolism and cholesterol metabolism were enriched in both genetic types, although phenotypic differences in plasma cholesterol suggest greater activation of this process in IB pigs. These results contribute to the understanding of molecular mechanisms driving phenotypic differences observed in IB and IBxDU pigs. Moreover, findings regarding lipid metabolism regulation might be of interest in research related to metabolic alterations in other species such as humans.

Also, we identified TRF that potentially regulate the observed transcriptomic differences. The different employed approaches allow highlighting several TRF especially interesting such as *CEBPs*, *EGRs*, *ATFs*, *PPARGC1B*, *FOXOs*, *TRIM63*, *MEFD2D*, *MYOD1* or *MYOG*.

Finally, genetic structural variations that might be associated to changes in expression and protein function were identified. Differentially segregating SNPs in IB and IBxDU piglets associated to those TRF that were more consistently identified were of special interest. Among them, *PPARGC1B* and *TRIM63* showed non-synonymous variants that might differentially regulate their function in IB and IBxDU pigs. Taken together, results found in the present study provide information about candidate genes and genetic polymorphisms potentially involved in phenotypic differences between IB and IBxDU associated to meat quality and production traits. Further validation of these genes and polymorphisms would contribute to their use in future breeding programs.

## Supporting Information

S1 Table Gene information, primer sequences, amplicon size and efficiency of genes selected for qPCR validation. (XLSX)

S2 Table Genes found differentially expressed between purebred (IB) and Duroc-crossbred (IBxDU) newborn pigs. (XLSX)

S3 Table RNA-Seq and qPCR validation results and correlation coefficient (r) between the two used methodologies. (XLSX)

S4 Table Enriched biological functions identified by IPA software in purebred (IB) and Duroc-crossbred (IBxDU) Iberian pigs (*p*<0.01). (XLSX)

S5 Table Canonical pathways enriched in IB and IBxDU animals based on DE genes and TFR identified either by IPA or by RIFs study. (XLSX)

S6 Table Structural variants identified in 14 candidate genes (*EGR2*, *FOS*, *FOXO1*, *FOXO3*, *IRF1*, *NOS1*, *TRIM63*, *ABRA*, *ATF3*, *TP53*, *HOXA9*, *STAT5B*, *ATF4* and *PPARGC1B)*. (XLSX)

## References

[1] Chang K, Da Costa N, Blackley R, Southwood O, Evans G, Plastow G, et al Relationships of myosin heavy chain fibre types to meat quality traits in traditional and modern pigs. Meat Science. 2003;64(1):93–103. 22062667 10.1016/s0309-1740(02)00208-5

[2] Astiz S, Gonzalez‐Bulnes A, Astiz I, Barbero A, Perez‐Solana M, Garcia‐Real I. Advanced onset of puberty after metformin therapy in swine with thrifty genotype. Experimental physiology. 2014;99(9):1241–52. 10.1113/expphysiol.2014.081455 25085845

[3] Murgiano L, D’Alessandro A, Egidi MG, Crisa A, Prosperini G, Timperio AM, et al Proteomics and transcriptomics investigation on longissimus muscles in Large White and Casertana pig breeds. Journal of proteome research. 2010;9(12):6450–66. 10.1021/pr100693h 20968299

[4] López-Bote C. Sustained utilization of the Iberian pig breed. Meat science. 1998;49:S17–S27. 22060709

[5] Muñoz G, Ovilo C, Silió L, Tomás A, Noguera J, Rodríguez M. Single-and joint-population analyses of two experimental pig crosses to confirm quantitative trait loci on chromosome 6 and leptin receptor effects on fatness and growth traits. Journal of animal science. 2009;87(2):459–68. 10.2527/jas.2008-1127 18952727

[6] Ovilo C, Fernández A, Noguera J, Barragán C, Letón R, Rodríguez C, et al Fine mapping of porcine chromosome 6 QTL and LEPR effects on body composition in multiple generations of an Iberian by Landrace intercross. Genetical research. 2005;85(01):57–67. 16089036 10.1017/s0016672305007330

[7] Fernandez-Figares I, Lachica M, Nieto R, Rivera-Ferre M, Aguilera J. Serum profile of metabolites and hormones in obese (Iberian) and lean (Landrace) growing gilts fed balanced or lysine deficient diets. Livestock Science. 2007;110(1):73–81.

[8] Torres-Rovira L, Astiz S, Caro A, Lopez-Bote C, Ovilo C, Pallares P, et al Diet-induced swine model with obesity/leptin resistance for the study of metabolic syndrome and type 2 diabetes. The Scientific World Journal. 2012;2012. 10.1100/2012/510149 PMC3354447 22629144

[9] Ventanas S, Ventanas J, Jurado A, Estevez M. Quality traits in muscle biceps femoris and back-fat from purebred Iberian and reciprocal Iberian x Duroc crossbred pigs. Meat Science. 2006;73(4):651–9. 10.1016/j.meatsci.2006.03.009 . 22062566

[10] Wood JD, Enser M, Fisher AV, Nute GR, Sheard PR, Richardson RI, et al Fat deposition, fatty acid composition and meat quality: A review. Meat Science. 2008;78(4):343–58. 10.1016/j.meatsci.2007.07.019 . 22062452

[11] Sepe A, Tchkonia T, Thomou T, Zamboni M, Kirkland JL. Aging and regional differences in fat cell progenitors–a mini-review. Gerontology. 2011;57(1):66–75. 10.1159/000279755 20110661 PMC3031153

[12] Gregoire FM, Smas CM, Sul HS. Understanding adipocyte differentiation. Physiol Rev. 1998;78(3):783–809. Epub 1998/07/23. . 9674695 10.1152/physrev.1998.78.3.783

[13] Li W, Zhao S, Huang Y, Yang M, Pan H, Zhang X, et al Expression of lipogenic genes during porcine intramuscular preadipocyte differentiation. Research in Veterinary Science. 2012;93(3):1190–4. 10.1016/j.rvsc.2012.06.006 . 22795880

[14] Ovilo C, Benitez R, Fernandez A, Nunez Y, Ayuso M, Fernandez A, et al Longissimus dorsi transcriptome analysis of purebred and crossbred Iberian pigs differing in muscle characteristics. Bmc Genomics. 2014;15 10.1186/1471-2164-15-413 . PMC4070551 24885501

[15] Ropka-Molik K, Zukowski K, Eckert R, Gurgul A, Piorkowska K, Oczkowicz M. Comprehensive analysis of the whole transcriptomes from two different pig breeds using RNA-Seq method. Animal Genetics. 2014;45(5):674–84. 10.1111/age.12184 . 24961663

[16] Pérez-Enciso M, Ferraz AL, Ojeda A, López-Béjar M. Impact of breed and sex on porcine endocrine transcriptome: a bayesian biometrical analysis. Bmc Genomics. 2009;10(1):89. 19239697 10.1186/1471-2164-10-89 PMC2656523

[17] Wickramasinghe S, Cánovas A, Rincón G, Medrano JF. RNA-sequencing: a tool to explore new frontiers in animal genetics. Livestock Science. 2014;166:206–16.

[18] Marioni J, Mason C, Mane S, Stephens M, Gilad Y. RNA-seq: An assessment of technical reproducibility and comparison with gene expression arrays. Genome Research. 2008;18(9):1509–17. 10.1101/gr.079558.108 . 18550803 PMC2527709

[19] Qian X, Ba Y, Zhuang Q, Zhong G. RNA-Seq technology and its application in fish transcriptomics. Omics: a journal of integrative biology. 2014;18(2):98–110. 10.1089/omi.2013.0110 24380445 PMC3920896

[20] Ghosh M, Sodhi S, Song KD, Kim J, Mongre R, Sharma N, et al Evaluation of body growth and immunity‐related differentially expressed genes through deep RNA sequencing in the piglets of Jeju native pig and Berkshire. Animal genetics. 2015;46(3):255–64. 10.1111/age.12281 25752324

[21] Pérez-Montarelo D, Madsen O, Alves E, Rodríguez MC, Folch JM, Noguera JL, et al Identification of genes regulating growth and fatness traits in pig through hypothalamic transcriptome analysis. Physiological genomics. 2014;46(6):195–206. 10.1152/physiolgenomics.00151.2013 24280257 PMC3949103

[22] Puig-Oliveras A, Ramayo-Caldas Y, Corominas J, Estellé J, Pérez-Montarelo D, Hudson NJ, et al. Differences in muscle transcriptome among pigs phenotypically extreme for fatty acid composition. 2014. 10.1371/journal.pone.0099720 PMC4057286 24926690

[23] Esteve-Codina A, Kofler R, Palmieri N, Bussotti G, Notredame C, Pérez-Enciso M. Exploring the gonad transcriptome of two extreme male pigs with RNA-seq. BMC genomics. 2011;12(1):552. 22067327 10.1186/1471-2164-12-552 PMC3221674

[24] Te Pas MF, Keuning E, Van De Wiel DJ, Young JF, Oksbjerg N, Kruijt L. Proteome profiles of Longissimus and Biceps femoris porcine muscles related to exercise and resting. Journal of Life Science. 2011;5:598–608.

[25] Karlsson A, Enfält A-C, Essén-Gustavsson B, Lundström K, Rydhmer L, Stern S. Muscle histochemical and biochemical properties in relation to meat quality during selection for increased lean tissue growth rate in pigs. Journal of Animal Science. 1993;71(4):930–8. 8478293 10.2527/1993.714930x

[26] Lefaucheur L, Lebret B, Ecolan P, Louveau I, Damon M, Prunier A, et al Muscle characteristics and meat quality traits are affected by divergent selection on residual feed intake in pigs. Journal of animal science. 2011;89(4):996–1010. 10.2527/jas.2010-3493 21148787

[27] Ayuso M, Fernández A, Isabel B, Rey A, Benítez R, Daza A, et al Long term vitamin A restriction improves meat quality parameters and modifies gene expression in Iberian pigs. Journal of Animal Science. 2015. 10.2527/jas.2014-8573 26115261

[28] Herault F, Vincent A, Dameron O, Le Roy P, Cherel P, Damon M. The longissimus and semimembranosus muscles display marked differences in their gene expression profiles in pig. 2014. 10.1371/journal.pone.0096491 PMC4014511 24809746

[29] Zhao X, Mo D, Li A, Gong W, Xiao S, Zhang Y, et al Comparative analyses by sequencing of transcriptomes during skeletal muscle development between pig breeds differing in muscle growth rate and fatness. PloS one. 2011;6(5):e19774 10.1371/journal.pone.0019774 21637832 PMC3102668

[30] Segura J, López-Bote CJ. A laboratory efficient method for intramuscular fat analysis. Food Chem. 2014;145:821–5. Epub 2013/10/17. S0308-8146(13)01233-8 [pii] 10.1016/j.foodchem.2013.08.131 . 24128551

[31] López-Bote CJ, Rey AI, Isabel B, Sanz R. Effect of feeding diets high in monounsaturated fatty acids and alpha-tocopheryl acetate to rabbits on resulting carcass fatty acid profile and lipid oxidation. Anim Sci. 1997;64:177–86.

[32] Cánovas A, Rincón G, Bevilacqua C, Islas-Trejo A, Brenaut P, Hovey RC, et al Comparison of five different RNA sources to examine the lactating bovine mammary gland transcriptome using RNA-Sequencing. Scientific reports. 2014;4. 10.1038/srep05297 PMC5381611 25001089

[33] Trapnell C, Williams BA, Pertea G, Mortazavi A, Kwan G, Van Baren MJ, et al Transcript assembly and quantification by RNA-Seq reveals unannotated transcripts and isoform switching during cell differentiation. Nature biotechnology. 2010;28(5):511–5. 10.1038/nbt.1621 20436464 PMC3146043

[34] Robinson MD, McCarthy DJ, Smyth GK. edgeR: a Bioconductor package for differential expression analysis of digital gene expression data. Bioinformatics. 2010;26(1):139–40. 10.1093/bioinformatics/btp616 19910308 PMC2796818

[35] Kamburov A, Pentchev K, Galicka H, Wierling C, Lehrach H, Herwig R. ConsensusPathDB: toward a more complete picture of cell biology. Nucleic acids research. 2011;39(suppl 1):D712–D7. 21071422 10.1093/nar/gkq1156 PMC3013724

[36] Hudson NJ, Dalrymple BP, Reverter A. Beyond differential expression: the quest for causal mutations and effector molecules. BMC Genomics. 2012;13(1):356. 22849396 10.1186/1471-2164-13-356 PMC3444927

[37] Reverter A, Hudson NJ, Nagaraj SH, Pérez-Enciso M, Dalrymple BP. Regulatory impact factors: unraveling the transcriptional regulation of complex traits from expression data. Bioinformatics. 2010;26(7):896–904. 10.1093/bioinformatics/btq051 20144946

[38] Almudevar A, Klebanov LB, Qiu X, Salzman P, Yakovlev AY. Utility of correlation measures in analysis of gene expression. NeuroRx. 2006;3(3):384–95. 16815221 10.1016/j.nurx.2006.05.037 PMC3593386

[39] Ng PC, Henikoff S. SIFT: Predicting amino acid changes that affect protein function. Nucleic acids research. 2003;31(13):3812–4. 12824425 10.1093/nar/gkg509 PMC168916

[40] Adzhubei IA, Schmidt S, Peshkin L, Ramensky VE, Gerasimova A, Bork P, et al A method and server for predicting damaging missense mutations. Nature methods. 2010;7(4):248–9. 10.1038/nmeth0410-248 20354512 PMC2855889

[41] Vandesompele J, De Preter K, Pattyn F, Poppe B, Van Roy N, De Paepe A, et al Accurate normalization of real-time quantitative RT-PCR data by geometric averaging of multiple internal control genes. Genome biology. 2002;3(7):research0034 12184808 10.1186/gb-2002-3-7-research0034 PMC126239

[42] Steibel JP, Poletto R, Coussens PM, Rosa GJM. A powerful and flexible linear mixed model framework for the analysis of relative quantification RT-PCR data. Genomics. 2009;94(2):146–52. 10.1016/j.ygeno.2009.04.008 . 19422910

[43] Miron M, Woody OZ, Marcil A, Murie C, Sladek R, Nadon R. A methodology for global validation of microarray experiments. Bmc Bioinformatics. 2006;7(1):333. 16822306 10.1186/1471-2105-7-333 PMC1539027

[44] Fuentes V, Ventanas S, Ventanas J, Estevez M. The genetic background affects composition, oxidative stability and quality traits of Iberian dry-cured hams: Purebred Iberian versus reciprocal Iberian x Duroc crossbred pigs. Meat Science. 2014;96(2):737–43. 10.1016/j.meatsci.2013.10.010 . 24200565

[45] Robina A, Viguera J, Perez-Palacios T, Mayoral AI, Vivo JM, Guillen MT, et al Carcass and meat quality traits of Iberian pigs as affected by sex and crossbreeding with different Duroc genetic lines. Span J Agric Res. 2013;11(4):1057–67. 10.5424/sjar/2013114-4637 .

[46] Serrano M, Valencia D, Nieto M, Lázaro R, Mateos G. Influence of sex and terminal sire line on performance and carcass and meat quality of Iberian pigs reared under intensive production systems. Meat science. 2008;78(4):420–8. 10.1016/j.meatsci.2007.07.006 22062461

[47] Torres-Rovira L, Tarrade A, Astiz S, Mourier E, Perez-Solana M, De La Cruz P, et al Sex and breed-dependent organ development and metabolic responses in foetuses from lean and obese/leptin resistant swine. PloS one. 2013;8(7):1–9. 10.1371/journal.pone.0066728 PMC3720837 23935823

[48] Palinski W. Maternal–Fetal Cholesterol Transport in the Placenta Good, Bad, and Target for Modulation. Circulation research. 2009;104(5):569–71. 10.1161/CIRCRESAHA.109.194191 19286612

[49] Woollett LA. The origins and roles of cholesterol and fatty acids in the fetus. Current opinion in lipidology. 2001;12(3):305–12. 11353334 10.1097/00041433-200106000-00010

[50] Wadsack C, Tabano S, Maier A, Hiden U, Alvino G, Cozzi V, et al Intrauterine growth restriction is associated with alterations in placental lipoprotein receptors and maternal lipoprotein composition. American Journal of Physiology-Endocrinology and Metabolism. 2007;292(2):E476–E84. 17003234 10.1152/ajpendo.00547.2005

[51] Herrera E. Implications of dietary fatty acids during pregnancy on placental, fetal and postnatal development—a review. Placenta. 2002;23:S9–S19. 11978055 10.1053/plac.2002.0771

[52] Herrera E, Amusquivar E, Lopez-Soldado I, Ortega H. Maternal lipid metabolism and placental lipid transfer. Hormone Research in Paediatrics. 2006;65(Suppl. 3):59–64. 10.1159/000091507 16612115

[53] Coleman RA, Haynes EB. Microsomal and lysosomal enzymes of triacylglycerol metabolism in rat placenta. Biochem J. 1984;217:391–7. 6696738 10.1042/bj2170391 PMC1153229

[54] Szabo AJ, Grimaldi RD, de Lellis R. Triglyceride synthesis by the human placenta. American Journal of Obstetrics & Gynecology. 1973;115(2):263–6. 4691844

[55] Gonzalez-Bulnes A, Torres-Rovira L, Ovilo C, Astiz S, Gomez-Izquierdo E, Gonzalez-Añover P, et al Reproductive, endocrine and metabolic feto-maternal features and placental gene expression in a swine breed with obesity/leptin resistance. General and comparative endocrinology. 2012;176(1):94–101. 10.1016/j.ygcen.2011.12.038 22251656

[56] Luo X, Yu C, Fu C, Shi W, Wang X, Zeng C, et al Identification of the differentially expressed genes associated with familial combined hyperlipidemia using bioinformatics analysis. Molecular medicine reports. 2015;11(6):4032–8. 10.3892/mmr.2015.3263 25625967 PMC4394960

[57] Mata P, Alonso R, Ruíz-Garcia A, Díaz-Díaz JL, González N, Gijón-Conde T, et al Hiperlipidemia familiar combinada: documento de consenso. Atención Primaria. 2014;46(8):440–6. 10.1016/j.aprim.2014.04.013 25034722 PMC6985613

[58] Granucci F, Petralia F, Urbano M, Citterio S, Di Tota F, Santambrogio L, et al The scavenger receptor MARCO mediates cytoskeleton rearrangements in dendritic cells and microglia. Blood. 2003;102(8):2940–7. 12842997 10.1182/blood-2002-12-3651

[59] Kabir SM, Lee E-S, Son D-S. Chemokine network during adipogenesis in 3T3-L1 cells: Differential response between growth and proinflammatory factor in preadipocytes vs. adipocytes. Adipocyte. 2014;3(2):97–106. 10.4161/adip.28110 24719782 PMC3979886

[60] Annayev Y, Adar S, Chiou Y-Y, Lieb JD, Sancar A, Ye R. Gene model 129 (Gm129) encodes a novel transcriptional repressor that modulates circadian gene expression. Journal of Biological Chemistry. 2014;289(8):5013–24. 10.1074/jbc.M113.534651 24385426 PMC3931061

[61] Merbitz-Zahradnik T, Wolf E. How is the inner Circadian Clock controlled by interactive clock proteins? FEBS letters. 2015. 10.1016/j.febslet.2015.05.024 25999309

[62] Fonken LK, Nelson RJ. The effects of light at night on circadian clocks and metabolism. Endocrine reviews. 2014;35(4):648–70. 10.1210/er.2013-1051 24673196

[63] Shimba S, Ogawa T, Hitosugi S, Ichihashi Y, Nakadaira Y, Kobayashi M, et al Deficient of a clock gene, brain and muscle Arnt-like protein-1 (BMAL1), induces dyslipidemia and ectopic fat formation. PloS one. 2011;6(9):e25231 10.1371/journal.pone.0025231 21966465 PMC3178629

[64] Thompson MR, Xu D, Williams BR. ATF3 transcription factor and its emerging roles in immunity and cancer. Journal of molecular medicine. 2009;87(11):1053–60. 10.1007/s00109-009-0520-x 19705082 PMC2783469

[65] Hai T, Hartman MG. The molecular biology and nomenclature of the activating transcription factor/cAMP responsive element binding family of transcription factors: activating transcription factor proteins and homeostasis. Gene. 2001;273(1):1–11. 11483355 10.1016/s0378-1119(01)00551-0

[66] Francesc R, Joan V, Elayne H, Marta G, Francesc V. FGF21 expression and release in muscle cells: involvement of MyoD and regulation by mitochondria-driven signalling. Biochemical Journal. 2014;463(2):191–9. 10.1042/BJ20140403 25055037

[67] Lin H-Q, Choi R, Chan K-L, Ip D, Tsim KW-k, Wan DC-c. Differential gene expression profiling on the muscle of acetylcholinesterase knockout mice: A preliminary analysis. Chemico-biological interactions. 2010;187(1):120–3. 20381477 10.1016/j.cbi.2010.03.054

[68] Stevenson EJ, Giresi PG, Koncarevic A, Kandarian SC. Global analysis of gene expression patterns during disuse atrophy in rat skeletal muscle. The Journal of physiology. 2003;551(1):33–48. 12844509 10.1113/jphysiol.2003.044701 PMC2343139

[69] Wallberg-Henriksson H, Zierath JR. GLUT4: a key player regulating glucose homeostasis? Insights from transgenic and knockout mice. Molecular membrane biology. 2001;18(3):205–11. 11681787 10.1080/09687680110072131

[70] Wang Y, Hudak C, Sul HS. Role of preadipocyte factor 1 in adipocyte differentiation. Clinical lipidology. 2010;5(1):109–15. 20414356 10.2217/clp.09.80 PMC2856086

[71] Cagnazzo M, Te Pas M, Priem J, De Wit A, Pool M, Davoli R, et al Comparison of prenatal muscle tissue expression profiles of two pig breeds differing in muscle characteristics. Journal of animal science. 2006;84(1):1–10. 16361485 10.2527/2006.8411

[72] Zhang GH, Lu JX, Chen Y, Zhao YQ, Guo PH, Yang JT, et al Comparison of the adipogenesis in intramuscular and subcutaneous adipocytes from Bamei and Landrace pigs. Biochemistry and Cell Biology. 2014;92(4):259–67. 10.1139/bcb-2014-0019 24943241

[73] Hamam D, Ali D, Vishnubalaji R, Hamam R, Al-Nbaheen M, Chen L, et al microRNA-320/RUNX2 axis regulates adipocytic differentiation of human mesenchymal (skeletal) stem cells. Cell death & disease. 2014;5(10):e1499. 25356868 10.1038/cddis.2014.462 PMC4237271

[74] Wang Z, Gerstein M, Snyder M. RNA-Seq: a revolutionary tool for transcriptomics. Nature Reviews Genetics. 2009;10(1):57–63. 10.1038/nrg2484 19015660 PMC2949280

[75] Butler AA, Roith DL. CONTROL OF GROWTH BY THE SOMATROPIC AXIS: Growth Hormone and the Insulin-Like Growth Factors Have Related and Independent Roles 1. Annual review of physiology. 2001;63(1):141–64. 10.1146/annurev.physiol.63.1.141 11181952

[76] Gerfault V, Louveau I, Mourot J. The effect of GH and IGF-I on preadipocytes from Large White and Meishan pigs in primary culture. General and comparative endocrinology. 1999;114(3):396–404. 10336827 10.1006/gcen.1999.7271

[77] Wabitsch M, Hauner H, Heinze E, Teller WM. The role of growth hormone/insulin-like growth factors in adipocyte differentiation. Metabolism. 1995;44(10 Suppl 4):45–9. Epub 1995/10/01. . 7476311 10.1016/0026-0495(95)90220-1

[78] Iwasaki S, Miyake M, Hayashi S, Watanabe H, Nagasawa Y, Terada S, et al Effect of Myostatin on Chemokine Expression in Regenerating Skeletal Muscle Cells. Cells Tissues Organs. 2013;198(1):66–74. 10.1159/000351462 23838214

[79] Posey AD, Swanson KE, Alvarez MG, Krishnan S, Earley JU, Band H, et al EHD1 mediates vesicle trafficking required for normal muscle growth and transverse tubule development. Developmental biology. 2014;387(2):179–90. 10.1016/j.ydbio.2014.01.004 24440153 PMC3987670

[80] Frateschi S, Keppner A, Malsure S, Iwaszkiewicz J, Sergi C, Merillat A-M, et al Mutations of the serine protease CAP1/Prss8 lead to reduced embryonic viability, skin defects, and decreased ENaC activity. The American journal of pathology. 2012;181(2):605–15. 10.1016/j.ajpath.2012.05.007 22705055

[81] Moinard C, Le Plenier S, Noirez P, Morio B, Bonnefont-Rousselot D, Kharchi C, et al Citrulline Supplementation Induces Changes in Body Composition and Limits Age-Related Metabolic Changes in Healthy Male Rats. The Journal of Nutrition. 2015:jn200626. 10.3945/jn.114.200626 26019250

[82] Bredt DS. Nitric oxide signaling specificity—the heart of the problem. Journal of cell science. 2003;116(1):9–15. 12456711 10.1242/jcs.00183

[83] D’Andrea M, Dal Monego S, Pallavicini A, Modonut M, Dreos R, Stefanon B, et al Muscle transcriptome profiling in divergent phenotype swine breeds during growth using microarray and RT‐PCR tools. Animal genetics. 2011;42(5):501–9. 10.1111/j.1365-2052.2010.02164.x 21906101

[84] Kim N-K, Park H-R, Lee H-C, Yoon D, Son E-S, Kim Y-S, et al Comparative studies of skeletal muscle proteome and transcriptome profilings between pig breeds. Mammalian Genome. 2010;21(5–6):307–19. 10.1007/s00335-010-9264-8 20532784

[85] Jaitovich A, Angulo M, Lecuona E, Dada LA, Welch LC, Cheng Y, et al High CO2 Levels Cause Skeletal Muscle Atrophy via AMP-activated Kinase (AMPK), FoxO3a Protein, and Muscle-specific Ring Finger Protein 1 (MuRF1). Journal of Biological Chemistry. 2015;290(14):9183–94. 10.1074/jbc.M114.625715 25691571 PMC4423704

[86] Damon M, Wyszynska-Koko J, Vincent A, Herault F, Lebret B. Comparison of muscle transcriptome between pigs with divergent meat quality phenotypes identifies genes related to muscle metabolism and structure. PLoS One. 2012;7(3):e33763 10.1371/journal.pone.0033763 22470472 PMC3312351

[87] Rivera-Ferre M, Aguilera J, Nieto R. Muscle fractional protein synthesis is higher in Iberian than in Landrace growing pigs fed adequate or lysine-deficient diets. The Journal of nutrition. 2005;135(3):469–78. 15735080 10.1093/jn/135.3.469

[88] Koc M, Mayerová V, Kračmerová J, Mairal A, Mališová L, Štich V, et al Stress of endoplasmic reticulum modulates differentiation and lipogenesis of human adipocytes. Biochemical and biophysical research communications. 2015;460(3):684–90. 10.1016/j.bbrc.2015.03.090 25813485

[89] Zheng Z, Zhang C, Zhang K. Role of unfolded protein response in lipogenesis. World journal of hepatology. 2010;2(6):203 10.4254/wjh.v2.i6.203 21160998 PMC2999286

[90] Pajukanta P, Lilja HE, Sinsheimer JS, Cantor RM, Lusis AJ, Gentile M, et al Familial combined hyperlipidemia is associated with upstream transcription factor 1 (USF1). Nature genetics. 2004;36(4):371–6. 14991056 10.1038/ng1320

[91] Pushpakom SP, Owen A, Back DJ, Pirmohamed M. RXRγ gene variants are associated with HIV lipodystrophy. Pharmacogenetics and genomics. 2013;23(8):438–41. 10.1097/FPC.0b013e328362cfc6 23759678

[92] Becker B, Misfeldt M. Evaluation of the mitogen-induced proliferation and cell surface differentiation antigens of lymphocytes from pigs 1 to 30 days of age. Journal of animal science. 1993;71(8):2073–8. 8376231 10.2527/1993.7182073x

[93] Sutherland M, Rodriguez-Zas S, Ellis M, Salak-Johnson J. Breed and age affect baseline immune traits, cortisol, and performance in growing pigs. Journal of animal science. 2005;83(9):2087–95. 16100063 10.2527/2005.8392087x

[94] Bergman I-M, Rosengren JK, Edman K, Edfors I. European wild boars and domestic pigs display different polymorphic patterns in the Toll-like receptor (TLR) 1, TLR2, and TLR6 genes. Immunogenetics. 2010;62(1):49–58. 10.1007/s00251-009-0409-4 19953243

[95] Amaral AJ, Ferretti L, Megens H-J, Crooijmans R, Nie H, Ramos-Onsins SE, et al Genome-wide footprints of pig domestication and selection revealed through massive parallel sequencing of pooled DNA. PloS one. 2011;6(4):e14782 10.1371/journal.pone.0014782 21483733 PMC3070695

[96] Balasubramanyam A. The role of the immune system in obesity and insulin resistance. Journal of obesity. 2013;2013. 10.1155/2013/616193 PMC3618935 23577240

[97] Exley MA, Hand L, O'Shea D, Lynch L. Interplay between the immune system and adipose tissue in obesity. Journal of Endocrinology. 2014;223(2):R41–R8. 10.1530/JOE-13-0516 25228503

[98] Xing K, Zhu F, Zhai L, Liu H, Wang Y, Wang Z, et al Integration of Transcriptome and Whole Genomic Resequencing Data to Identify Key Genes Affecting Swine Fat Deposition. 2015. 10.1371/journal.pone.0122396 PMC4388518 25849573

[99] Veum V, Dankel S, Gjerde J, Nielsen H, Solsvik M, Haugen C, et al The nuclear receptors NUR77, NURR1 and NOR1 in obesity and during fat loss. International Journal of Obesity. 2012;36(9):1195–202. 10.1038/ijo.2011.240 22143616

[100] Boyle KB, Hadaschik D, Virtue S, Cawthorn WP, Ridley SH, O'Rahilly S, et al The transcription factors Egr1 and Egr2 have opposing influences on adipocyte differentiation. Cell Death & Differentiation. 2009;16(5):782–9. 19229250 10.1038/cdd.2009.11 PMC2670277

[101] Berkers CR, Maddocks OD, Cheung EC, Mor I, Vousden KH. Metabolic regulation by p53 family members. Cell metabolism. 2013;18(5):617–33. 10.1016/j.cmet.2013.06.019 23954639 PMC3824073

[102] Vousden KH, Ryan KM. p53 and metabolism. Nature Reviews Cancer. 2009;9(10):691–700. 10.1038/nrc2715 19759539

[103] Hannenhalli S, Kaestner KH. The evolution of Fox genes and their role in development and disease. Nature Reviews Genetics. 2009;10(4):233–40. 10.1038/nrg2523 19274050 PMC2733165

[104] Wijchers P, Burbach J, Smidt M. In control of biology: of mice, men and Foxes. Biochem J. 2006;397:233–46. 16792526 10.1042/BJ20060387 PMC1513289

[105] Gupta D, Leahy AA, Monga N, Peshavaria M, Jetton TL, Leahy JL. Peroxisome Proliferator-activated Receptor γ (PPARγ) and Its Target Genes Are Downstream Effectors of FoxO1 Protein in Islet β-Cells MECHANISM OF β-CELL COMPENSATION AND FAILURE. J Biol Chem. 2013;288(35):25440–9. 10.1074/jbc.M113.486852 23788637 PMC3757206

[106] Allen DL, Unterman TG. Regulation of myostatin expression and myoblast differentiation by FoxO and SMAD transcription factors. American Journal of Physiology-Cell Physiology. 2007;292(1):C188–C99. 16885393 10.1152/ajpcell.00542.2005

[107] Hakuno F, Yamauchi Y, Kaneko G, Yoneyama Y, Nakae J, Chida K, et al Constitutive expression of insulin receptor substrate (IRS)-1 inhibits myogenic differentiation through nuclear exclusion of Foxo1 in L6 myoblasts. PloS one. 2011;6(10):e25655 10.1371/journal.pone.0025655 21991327 PMC3185002

[108] Handschin C, Spiegelman BM. Peroxisome proliferator-activated receptor gamma coactivator 1 coactivators, energy homeostasis, and metabolism. Endocr Rev. 2006;27(7):728–35. Epub 2006/10/05. 10.1210/er.2006-0037 . 17018837

[109] Demmerle J, Koch AJ, Holaska JM. Emerin and histone deacetylase 3 (HDAC3) cooperatively regulate expression and nuclear positions of MyoD, Myf5, and Pax7 genes during myogenesis. Chromosome Research. 2013;21(8):765–79. 10.1007/s10577-013-9381-9 24062260 PMC4829207

[110] Edmondson DG, Cheng T, Cserjesi P, Chakraborty T, Olson EN. Analysis of the myogenin promoter reveals an indirect pathway for positive autoregulation mediated by the muscle-specific enhancer factor MEF-2. Molecular and cellular biology. 1992;12(9):3665–77. 1324403 10.1128/mcb.12.9.3665-3677.1992 PMC360220

[111] Ivana L, Ohkawa Y, Berkes CA, Bergstrom DA, Dacwag CS, Tapscott SJ, et al MyoD targets chromatin remodeling complexes to the myogenin locus prior to forming a stable DNA-bound complex. Molecular and cellular biology. 2005;25(10):3997–4009. 15870273 10.1128/MCB.25.10.3997-4009.2005 PMC1087700

[112] Nabeshima Y, Hanaoka K, Hayasaka M, Esuml E, Li S, Nonaka I, et al. Myogenin gene disruption results in perinatal lethality because of severe muscle defect. 1993. 10.1038/364532a0 8393146

[113] Shi X, Wallis AM, Gerard RD, Voelker KA, Grange RW, DePinho RA, et al Foxk1 promotes cell proliferation and represses myogenic differentiation by regulating Foxo4 and Mef2. Journal of cell science. 2012;125(22):5329–37. 22956541 10.1242/jcs.105239 PMC3561855

[114] Zhao Y, Li J, Liu H, Xi Y, Xue M, Liu W, et al Dynamic transcriptome profiles of skeletal muscle tissue across 11 developmental stages for both Tongcheng and Yorkshire pigs. BMC genomics. 2015;16(1):377. 25962502 10.1186/s12864-015-1580-7 PMC4437458

[115] Lee S-H, Lee S, Yang H, Song S, Kim K, Saunders TL, et al Notch pathway targets proangiogenic regulator Sox17 to restrict angiogenesis. Circulation research. 2014;115(2):215–26. 10.1161/CIRCRESAHA.115.303142 24755984

[116] Nishizawa S, Koya T, Ohno Y, Goto A, Ikuita A, Suzuki M, et al Regeneration of injured skeletal muscle in heat shock transcription factor 1‐null mice. Physiological reports. 2013;1(3):e00071 10.1002/phy2.71 24303143 PMC3835021

[117] Yasuhara K, Ohno Y, Kojima A, Uehara K, Beppu M, Sugiura T, et al Absence of heat shock transcription factor 1 retards the regrowth of atrophied soleus muscle in mice. Journal of Applied Physiology. 2011;111(4):1142–9. 10.1152/japplphysiol.00471.2011 21817109

[118] Uzun A, Leslin CM, Abyzov A, Ilyin V. Structure SNP (StSNP): a web server for mapping and modeling nsSNPs on protein structures with linkage to metabolic pathways. Nucleic acids research. 2007;35(suppl 2):W384–W92. 17537826 10.1093/nar/gkm232 PMC1933130

[119] Fan B, Lkhagvadorj S, Cai W, Young J, Smith R, Dekkers J, et al Identification of genetic markers associated with residual feed intake and meat quality traits in the pig. Meat Science. 2010;84(4):645–50. 10.1016/j.meatsci.2009.10.025 20374837

[120] Johnson AD, Zhang Y, Papp AC, Pinsonneault JK, Lim J-E, Saffen D, et al Polymorphisms affecting gene transcription and mRNA processing in pharmacogenetic candidate genes: detection through allelic expression imbalance in human target tissues. Pharmacogenetics and genomics. 2008;18(9):781 10.1097/FPC.0b013e3283050107 18698231 PMC2779843

[121] Verardo L, Nascimento C, Silva F, Gasparino E, Martins M, Toriyama E, et al Identification and validation of differentially expressed genes from pig skeletal muscle. Journal of Animal Breeding and Genetics. 2013;130(5):372–81. 10.1111/jbg.12006 24074174

[122] Chen SN, Czernuszewicz G, Tan Y, Lombardi R, Jin J, Willerson JT, et al Human molecular genetic and functional studies identify TRIM63, encoding Muscle RING Finger Protein 1, as a novel gene for human hypertrophic cardiomyopathy. Circul Res. 2012;111(7):907–19. 10.1161/CIRCRESAHA.112.270207 PMC3482312 22821932

[123] Villegas R, Williams SM, Gao YT, Long J, Shi J, Cai H, et al Genetic Variation in the Peroxisome Proliferator‐Activated Receptor (PPAR) and Peroxisome Proliferator‐Activated Receptor Gamma Co‐activator 1 (PGC1) Gene Families and Type 2 Diabetes. Annals of human genetics. 2014;78(1):23–32. 10.1111/ahg.12044 24359475 PMC3936468

[124] Franks PW, Christophi CA, Jablonski KA, Billings LK, Delahanty LM, Horton ES, et al Common variation at PPARGC1A/B and change in body composition and metabolic traits following preventive interventions: the Diabetes Prevention Program. Diabetologia. 2014;57(3):485–90. 10.1007/s00125-013-3133-4 24317794 PMC4154629

[125] Jacobs K, Rohrer G, Van Poucke M, Piumi F, Yerle M, Barthenschlager H, et al Porcine PPARGC1A (peroxisome proliferative activated receptor gamma coactivator 1A): coding sequence, genomic organization, polymorphisms and mapping. Cytogenetic and genome research. 2006;112(1–2):106–13. 16276098 10.1159/000087521

[126] Hulbert A, Pamplona R, Buffenstein R, Buttemer W. Life and death: metabolic rate, membrane composition, and life span of animals. Physiol Rev. 2007;87(4):1175–213. 17928583 10.1152/physrev.00047.2006

